# Identification of lysosome‐targeting drugs with anti‐inflammatory activity as potential invasion inhibitors of treatment resistant HER2 positive cancers

**DOI:** 10.1007/s13402-021-00603-2

**Published:** 2021-05-03

**Authors:** Malene Bredahl Hansen, Maria Postol, Siri Tvingsholm, Inger Ødum Nielsen, Tiina Naumanen Dietrich, Pietri Puustinen, Kenji Maeda, Christoffel Dinant, Robert Strauss, David Egan, Marja Jäättelä, Tuula Kallunki

**Affiliations:** 1 Cell Death and Metabolism, Center for Autophagy, Recycling and Disease, Danish Cancer Society Research Center, Strandboulevarden 49, 2100 Copenhagen, Denmark; 2 Genome Integrity Group, Center for Autophagy, Recycling and Disease, Danish Cancer Society Research Center, Strandboulevarden 49, 2100 Copenhagen, Denmark; 3 Core Facility for Bioimaging, Danish Cancer Society Research Center, Strandboulevarden 49, 2100 Copenhagen, Denmark; 4 Department of Cell Biology, University Medical Center Utrecht (UMCU), Utrecht, The Netherlands; 5 Present Address: Core Life Analytics, Padualaan, 83584 CH Utrecht The Netherlands; 6 Department of Cellular and Molecular Medicine, Faculty of Health and Medical Sciences, University of Copenhagen, 2200 Copenhagen, Denmark; 7 Department of Drug Design and Pharmacology, Faculty of Health and Medical Sciences, University of Copenhagen, 2200 Copenhagen, Denmark

**Keywords:** HER2/ErbB2, Lysosome targeting drug, Anti‐inflammatory activity, Lapatinib, Tumor spheroid, Tumor organoid, Invasive growth, Drug multipurposing

## Abstract

**Purpose:**

Most HER2 positive invasive cancers are either intrinsic non-responsive or develop resistance when treated with 1st line HER2 targeting drugs. Both 1st and 2nd line treatments of HER2 positive cancers are aimed at targeting the HER2 receptor directly, thereby strongly limiting the treatment options of HER2/ErbB2 inhibition resistant invasive cancers.

**Methods:**

We used phenotypic high throughput microscopy screening to identify efficient inhibitors of ErbB2-induced invasion using 1st line HER2 inhibitor trastuzumab- and pertuzumab-resistant, p95-ErbB2 expressing breast cancer cells in conjunction with the Prestwick Chemical Library®. The screening entailed a drug’s ability to inhibit ErbB2-induced, invasion-promoting positioning of lysosomes at the cellular periphery, a phenotype that defines their invasiveness. In addition, we used high throughput microscopy and biochemical assays to assess the effects of the drugs on lysosomal membrane permeabilization (LMP) and autophagy, two features connected to cancer treatment. Using 2nd line HER2 inhibitor lapatinib resistant 3-dimensional model systems, we assessed the effects of the drugs on ErbB2 positive breast cancer spheroids and developed a high-throughput invasion assay for HER2 positive ovarian cancer organoids for further evaluation.

**Results:**

We identified Auranofin, Colchicine, Monensin, Niclosamide, Podophyllotoxin, Quinacrine and Thiostrepton as efficient inhibitors of invasive growth of 2nd line HER2 inhibitor lapatinib resistant breast cancer spheroids and ovarian cancer organoids. We classified these drugs into four groups based on their ability to target lysosomes by inducing autophagy and/or LMP, i.e., drugs inducing early LMP, early autophagy with late LMP, late LMP, or neither.

**Conclusions:**

Our results indicate that targetable lysosome-engaging cellular pathways downstream of ErbB2 contribute to invasion. They support lysosomal trafficking as an attractive target for therapy aiming at preventing the spreading of cancer cells. Since these drugs additionally possess anti-inflammatory activities, they could serve as multipurpose drugs simultaneously targeting infection/inflammation and cancer spreading.

**Supplementary Information:**

The online version contains supplementary material available at 10.1007/s13402-021-00603-2.

## Introduction

Breast cancer is the second most common cause for cancer mortality in women. Its lethality is caused by highly invasive, metastatic and treatment-resistant cancer cells. HER2/ErbB2 positive breast cancer represents about 20 % of invasive breast cancers. Despite current efficient treatments, the majority of HER2 positive, advanced invasive breast cancers treated with 1st line HER2 targeting therapy (trastuzumab and/or pertuzumab) together with chemotherapy are either initially non-responsive or develop resistant disease [1–3]. The existing treatments against relapsed HER2 positive breast cancer (e.g., TDM1 and lapatinib) are, as the 1st line treatment modalities trastuzumab and pertuzumab, directly targeting HER2. Since resistance towards HER2 targeting can be expected [4, 5], and since the major challenge in HER2 positive cancer is its highly invasive nature, an alternative approach would be to develop therapies that target the invasion-promoting functions of HER2/ErbB2.

Lysosome-mediated invasion is promoted by ErbB2 signalling and is based on the ability of lysosomes to secrete their hydrolytic contents into the extracellular space to initiate and support invasion through a process called lysosomal exocytosis [6–8]. Lysosomes are specialized for intracellular degradation. In lysosomal exocytosis, lysosomes clear themselves by localizing to the vicinity of the plasma membrane, fusing their membrane with it and emptying their hydrolytic contents into the extracellular space. Cancer cells can highjack this basic cellular function and use it to initiate and promote degradation of the extracellular matrix. Secreted lysosomal hydrolases, especially cysteine cathepsin B, promote invasion by cleaving extracellular matrix components directly, or indirectly by initiating the activation of a cascade of matrix metalloproteases [8–10]. Supporting the role of lysosomes and lysosomal hydrolases in cancer invasion, it has been found that inhibition of different lysosomal hydrolases, especially cysteine cathepsins, prevents invasion and metastasis in various *in vivo* cancer models [11–15].

Lysosomes play a dual role in tumorigenesis. They can promote invasion and metastasis by emptying their contents into the extracellular space or, upon lysosomal membrane permeabilization (LMP), lysosomes can empty their contents into the cytosol to initiate and promote a caspase-independent form of programmed cell death known as “lysosome-dependent cell death” [8]. Several compounds that can induce LMP are under investigation as anti-cancer drugs (e.g., so called cationic amphiphilic drugs: CADs) [8, 16, 17]. Activation of HER2-induced, invasion promoting downstream signalling correlates with cancer cell sensitization to lysosome membrane permeabilization (LMP) [6, 8, 18], meaning that when lysosomes get ready for exocytosis, they have most likely also become more sensitive for permeabilization. Thus, directing lysosomes away from the cell membrane may be a way to improve the therapeutic outcome of LMP in these cells.

In this study we employed the Prestwick Chemical Library® with 1200 commonly used drugs for high-throughput screening of compounds that can redirect lysosomes of ErbB2 expressing, 1st line ErbB2-inhibitor resistant breast cancer cells from their invasion-promoting location near the plasma membrane to their less cancerous position at the perinuclear area. We hypothesize that engaging lysosomes into positions where they cannot undergo lysosomal exocytosis and secrete their contents into the extracellular space, can prevent their invasion-promoting function. We analysed the most efficient compounds for their ability to induce LMP, autophagy, cathepsin B inhibition and to inhibit invasion of lapatinib-resistant HER2 positive mammary cancer spheroids and HER2 positive, lapatinib-resistant ovarian cancer organoids using high-throughput confocal microscopy and digital image analysis. We found a promising repurposing potential for several compounds that are both inhibiting the invasion pathway downstream of ErbB2 and are able to maintain the activity of lysosomal hydrolase cathepsin B, which is needed for normal lysosomal function and known as the major executioner of lysosome-dependent cell death induced by common anti-cancer treatments [16].

## Materials and methods

### High‐throughput screen and establishment of the PNLA score

Cells were seeded at a density of 4000 cells/well in 96-well plates (4titude Vision #4ti-0221). At 50 % confluence, the cells were treated with 5 µM of each Prestwick Chemical Library® compound for 24 h. Two to four wells were used for positive and negative controls. Cells were fixed and stained for nuclei (Hoechst) and lysosomes (LAMP2), as described under “immunocytochemistry” (Supplementary file 1). The plates were imaged using ImageXpress Micro Confocal in widefield mode, using a 40x air objective. Hoechst immunostaining was captured using a DAPI filter and LAMP2 immunostaining using a FITC filter. Images were subjected to segmentation using the custom module editor in MetaXpress analysis software (Supplementary Fig. 1a). Nuclei were segmented using the “Find Round Objects” module and masked using an appropriate threshold from the DAPI filter images. The nuclei were reduced by two pixels (0.325 μm) and defined as “Nuclei”. The “Nuclei” were then expanded with 25 pixels (4.0625 μm) using the “Grow Objects Without Touching” module and defined as “Expanded Nuclei”. All bordering objects were removed. “Nuclei” were subtracted from “Expanded Nuclei” and the resulting rings were defined as “Perinuclear rings”. Lysosomes were segmented using the “Find Round Objects” module with an appropriate threshold from the FITC filter images and defined “Lysosomes”. An overlay of “Perinuclear ring” and “Lysosomes” was made, and the average area of a lysosome puncta within the “Perinuclear ring” was defined as the readout, termed the perinuclear lysosome accumulation (PNLA) score.

### *In vivo* tumor growth and organoid isolation

Female NOG mice 7 weeks of age were obtained from Taconic, Denmark. 1,5 million human high-grade serous ovarian cancer OVC316 cells were injected into the uppermost mammary fat pads of the mice, after which tumor diameters were measured twice a week. When the diameters reached 12 mm, the mice were sacrificed, and the tumors were collected. Next, they were chopped into pieces and homogenized using mechanical force. The resulting pieces were digested with trypsin for 10 min in a 37^o^C tissue culture incubator while shaking, after which they were incubated for 1 h in a collagenase solution (10 mg/ml, Sigma-Aldrich) in organoid growth medium (DMEM/MEGM; GIBCO/Lonza) supplemented with 5 % FBS (GIBCO), 0.25 % penicillin and streptomycin solution (GIBCO) and 5 µg/ml insulin (Sigma-Aldrich). Next, pieces were pelleted by centrifugation for 10 min at 1500 rpm and digested in a DNAse solution (10 µg/ml, Roche) in DMEM/MEGM for 5 min at RT. Single cells were separated from organoids by differential centrifugation.

### 3D organoid invasion assay and analysis

Freshly isolated organoids were embedded in Cultrex® GFR BME basement membrane matrix in a 1:1 dilution with organoid growth media. Organoids were seeded into 96-well microplates (4titude), incubated in a humidified 5 % CO2 tissue culture incubator at 37^o^C and imaged at day 1 and day 3 (d1 ,d3) timepoints using an ImageXpress micro-confocal high throughput microscope in widefield mode. Image analysis was performed by building a custom module analysis pipeline in MetaXpress, where the “Bottom Hat” filter was used to enhance the dark organoid areas in the transmitted light images. The “Auto Threshold” module was used to segment the bright organoid areas in the Bottom Hat filtered image. The “Dilate Filter” function was used to grow the bright organoid segmentations to close caps. The remaining holes were filled using the “Fill Holes” function. A filter mask with a minimum area threshold was used to exclude the smallest segmented areas that were not organoids. The total area covered by organoids was calculated per drug and timepoint and presented as % change, where 100 % change in the organoid size corresponds to doubling of the organoid size and 0 % indicates no change in organoid size.

### Statistics

Statistic analysis was performed using GraphPad Prism 7.0 and R software. Values are presented as mean ± SEM. Student’s t-test was used when two samples were compared and one-way ANOVA with Dunnett’s multiple comparisons test was used when the same control was compared to different samples. A *p* value > 0.05 was considered non-significant (ns), whereas statistical significance is indicated as * *p* < 0.05, ** *p* < 0.01, *** *p* < 0.001 and **** *p* < 0.0001. The Z-factor or Z´(Z-prime) for each plate was calculated using the formula Z´=$1-3\frac{{(\sigma }_{p+}{\sigma }_{n)}}{\left|{\mu }_{p }-{\mu }_{n}\right|}$, where µ is the mean and σ is the standard deviation of the positive (*p*) and negative (*n*) controls. Robust Z-score was used for data normalization: Robust ${Z}_{xi}$ =$\frac{{x}_{i -{median(X}_{all })}}{MAD\left({X}_{all}\right)}$, where ${x}_{i}$ is an individual data point for a drug, ${x}_{all}$ is the mean of all measurements in a plate and MAD is the median absolute deviation for the standard deviation.

A more detailed list of the materials and methods used is provided in Supplementary Information file 1.

## Results

### Identification of compounds that can change lysosome distribution in 1st line HER2 inhibitor treatment resistant breast cancer cells

**Fig. 1:**
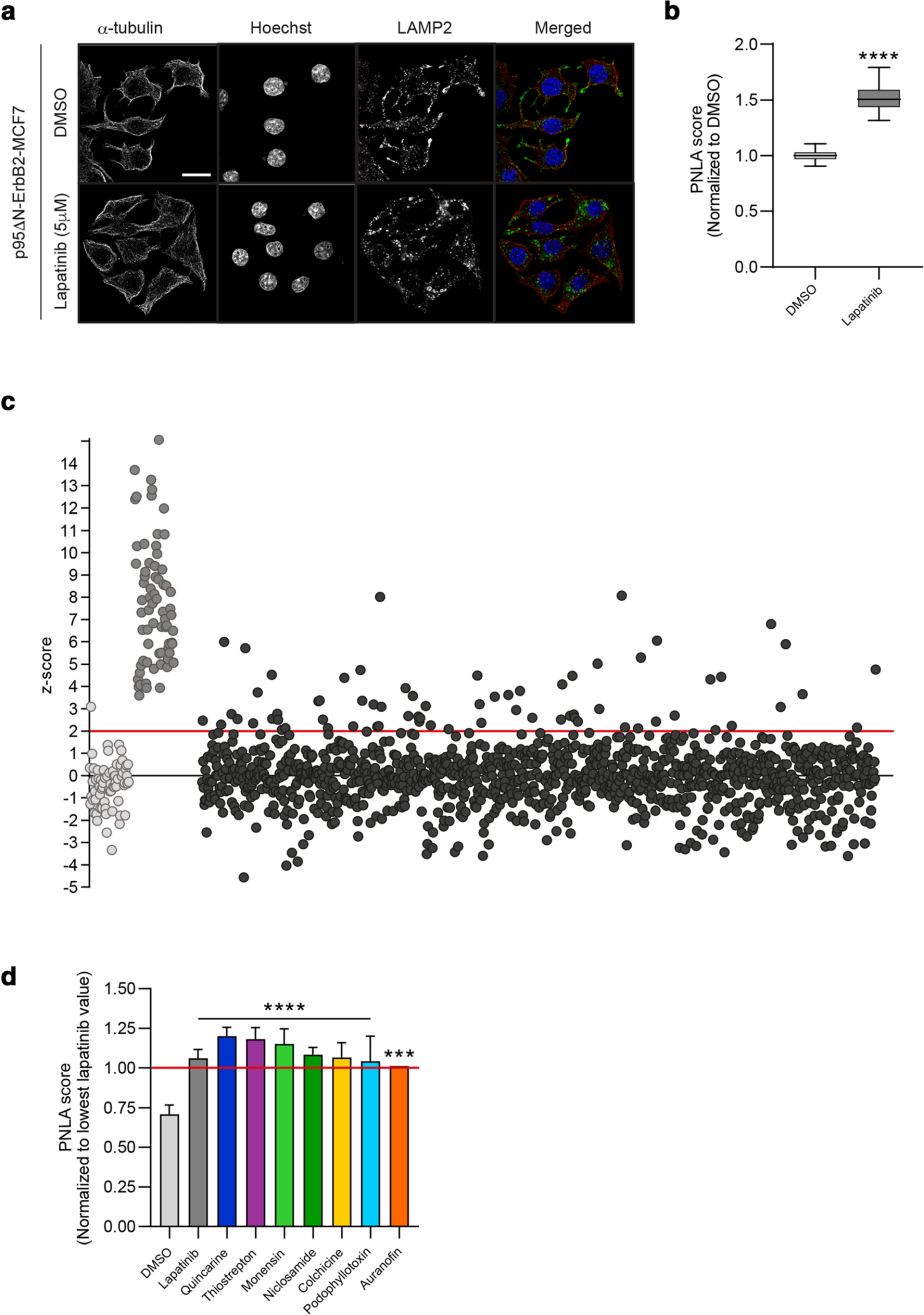
Identification of drugs that can change the positioning of lysosomes. **a** Effect of lapatinib-treatment to lysosome distribution. Representative immunofluorescence images of p95-ErbB2-MCF7 cells treated for 24 h with vehicle control (DMSO) or 5 µM lapatinib, fixed and stained with Hoechst for the detection of the nuclei (blue) and immune-stained for α-tubulin (red) for the detection of cytoskeleton and LAMP2 (green) for lysosomes. Scalebar: 20 μm. **b** Quantification LAMP2 staining in the perinuclear area of DMSO- and lapatinib-treated p95-ErbB2-MCF7 cells (24 h). The images were acquired using an ImageXpress microconfocal microscope with 40x objective. Analyses were performed using MetaXpress software. The perinuclear lysosome accumulation scores (PNLA scores) for DMSO and lapatinib (5 µM) are presented. Unpaired, parametric t-test. ****p < 0.0001 of a triplicate experiment of three biological replicates. **c** Screening for compounds that accumulate pericellular lysosomes to a perinuclear position. Cells were treated for 24 h with 5 µM Prestwick Chemical Library drugs, fixed and stained for nucleus and lysosomes as in 1a. Images were acquired at 24 h after treatment using an ImageXpress microconfocal system with 40x objective. Analyses were performed using MetaXpress software. Dot plot of DMSO (light gray dots), lapatinib (dark gray dots) and Prestwick Chemical Library® drugs (black dots) based on their Z-scores. Compounds with a Z-score of 2 or higher (2 median absolute deviations from the median of the sample population; red line) were selected for a secondary screen (n = 1). **d** Quantification of the best hits from the secondary screen. Column graph of hits from the secondary screen that had an equal or higher PNLA score than lapatinib. Values are normalized to the lowest detected value for the lapatinib treatment (red line) (n = 1–3). Statistics by ANOVA

Induction of expression of the constitutively active N-terminally truncated 95 kD form of HER2/ErbB2 (p95-ErbB2) renders MCF7 breast cancer cells invasive [6, 19]. The invasion-promoting, ErbB2-induced distribution of lysosomes to the cellular periphery can be efficiently reversed with 24 h treatment of 5 µM HER2/ERbB2 inhibitor lapatinib (Fig. 1a) [20, 21]. These cells are non-responsive to the standard ErbB2-targeting clinical antibodies trastuzumab and pertuzumab, since p95-ErbB2 lacks their binding sites [22]. This turns them into a unique *in vitro* model to mimic trastuzumab/pertuzumab-resistant p95-ErbB2 expressing breast cancer, which comprises ~ 25 % of HER2 positive, treatment-resistant breast cancers. Their responsiveness to lapatinib defines them as a model of breast cancer that has become resistant to standard 1st line treatment, but is still treatable with lapatinib, to which it is expected to develop resistance in time.

We used p95-ErbB2-MCF7 cells to set up a high-throughput microscopy screen to identify drugs that can reverse their ErbB2-induced, invasion-promoting lysosomal distribution at the cellular periphery. We aimed to identify drugs that could be used as monotherapy or as support of existing ErbB2 inhibitors for treatment-resistant cases. We used 24 h treatment with DMSO or lapatinib as respective negative and positive controls to set up screening conditions using images acquired with an ImageXpress Micro Confocal automated microscope and their quantification for the appearance of lysosomes at the perinuclear area (Supplementary Fig. 1a). The difference in lysosome distribution between lapatinib and DMSO was found to be significant (Fig. 1b). We used this system to define the perinuclear lysosome accumulation score (PNLA score) for the evaluation of the extent of accumulation. A high-throughput screen with compounds from the Prestwick Chemical Library® was set up by treating cells for 24 h with each of them (5 µM), followed by cell fixing and staining (Supplementary Data Set 1). The Z-factor for each plate was calculated utilizing DMSO and lapatinib. Only plates with a positive Z-factor score were included in the screen results by following the acceptable Z-factor recommendation (0 < Z < 0.5) to be used for complex phenotypic screening assays [23]. Robust Z scores were calculated for each drug, and drugs with a robust Z score of two or higher were analysed further (Fig. 1c). The results represent a pool of results from 24 different plates. Thus, the Z scores for lapatinib are highest on plates with no positive hits, making some of them higher than for the screened compounds (Fig. 1c). We set up a secondary screen with compounds with a Z score of two or higher and reasoned that some of them may inhibit invasion efficiently, but differently from lapatinib. The histograms for the Gaussian distribution of the Z score (Supplementary Fig. 1b) and the cell count (Supplementary Fig. 1c) were in a normal range as is required for a reliable Z score calculation.

Seven of the 64 re-screened compounds were found to be equal or more efficient than lapatinib in translocating lysosomes to the perinuclear area (Fig. 1d). These were Auranofin, Colchicine, Monensin, Niclosamide, Podophyllotoxin, Quinacrine and Thiostrepton. Six of these also efficiently reversed the ErbB2-induced “invasive” distribution of lysosomes in SKOV3ip1 ovarian cancer cells (Supplementary Fig. 1d).

### Lysosome‐dependent cell death and perinuclear lysosome distribution

**Fig. 2:**
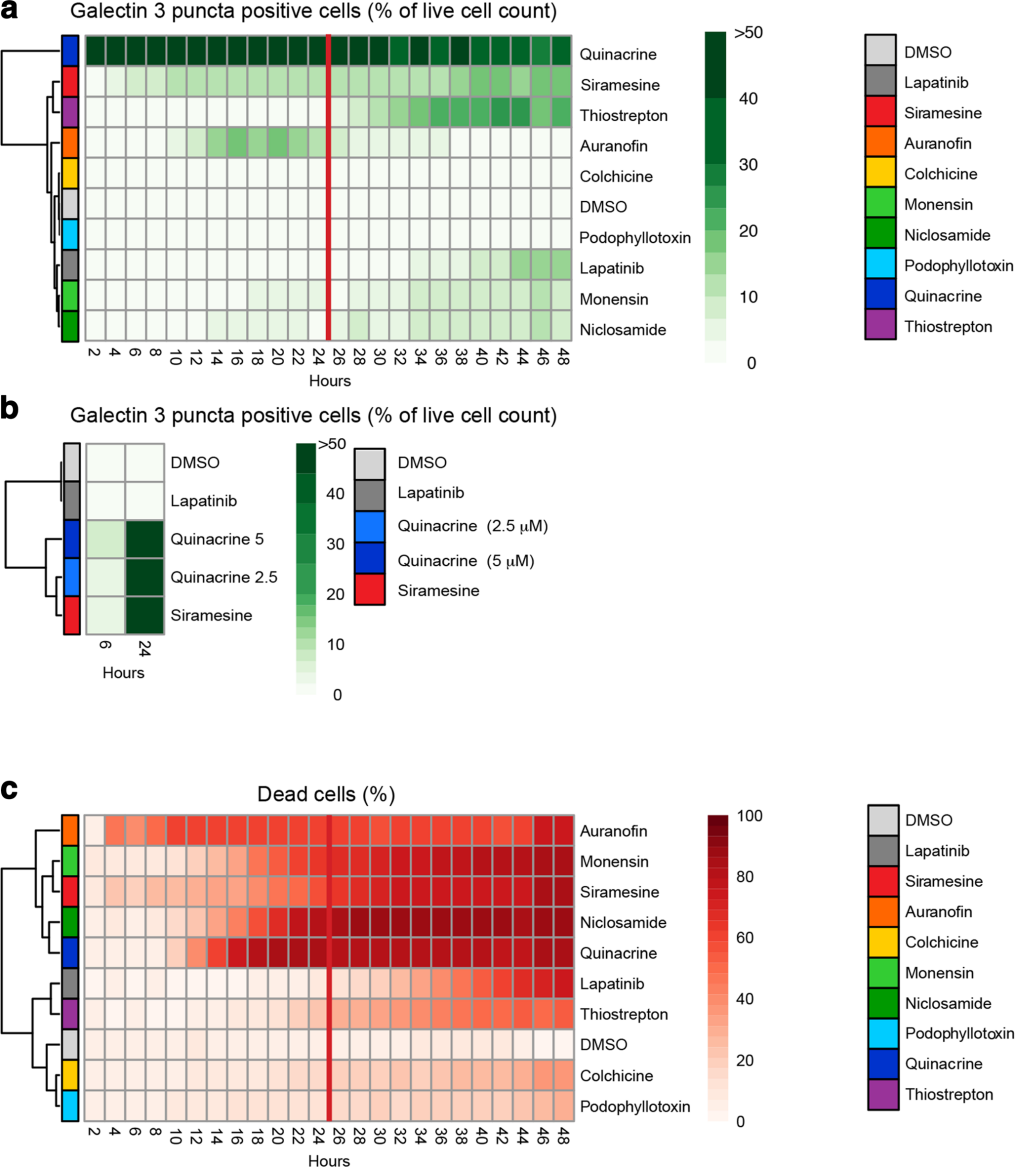
Detection of lysosome membrane permeabilization (LMP) and cell death. **a** Rate and efficiency of drug-induced formation of EGFP-Gal3 puncta. Heatmap of puncta detection in EGFP-Gal3-MCF7 cells showing % of mean of the EGFP-Gal3 puncta-positive live cells treated with 5 µM of the indicated compounds and followed up to 48 h. The images were acquired at 2 h intervals using an ImageXpress microconfocal system with 40x objective. Analyses were performed using MetaXpress software. A cell was considered EGFP-Gal3 puncta positive if 3 or more puncta were detected. The mean represents a mean of three biological replicates done in triplicate wells. 8 µM Siramesine was used as a positive control. Red line points at the 24 h timepoint that was used in the calculation of the PNLA scores in 1d. **b** Gal3 puncta formation by the autofluorescent drug Quinacrine. Gal3 puncta formation in fixed and Gal3 immunostained parental MCF7 cells treated for 6 h and 24 h with DMSO, 5 µM lapatinib, 8 µM Siramesine and both 2.5 and 5 µM concentrations of Quinacrine. The values represent means of three biological replicates done as three technical repeats. **c** Variation of cell death induction in EGFP-Gal3-MCF7 cells treated for 24 h with 5 µM of the indicated compounds. Heatmap presentation of the percent of cell death for each treatment using the information subtracted from images used in 2a. The percentage of dead cells was calculated from the total amount of cells detected in each site. Dead cell detection was done by determining high intensity nuclear stain signals (shrinking nucleus) and using transmitted light images. The cells that were not scored as dead cells were calculated as live cells. The mean values shown represent a mean of three biological replicates done in triplicate wells

To assess whether the different treatments can cause lysosomal membrane permeabilization (LMP), we used live imaging of MCF7 cells stably expressing pEGFP-Galectin 3 (pEGFP-Gal3) [24, 25], which we treated with 5 µM of the respective drugs or 8 µM of the well-known LMP inducing drug Siramesine, which is not included in the library, as a positive control (Supplementary Data Set 1). The induction of LMP was visualized and quantified as formation of EGFP-Gal3 puncta at the lysosomal membrane as a response to leaking lysosomes, utilizing the ability of Gal3 to accumulate into the sites of leakage [26]. We found that only Auranofin and Siramesine caused appearance of EGFP-Gal3 puncta before the 12 h time point (Fig. 2a). With Niclosamide and Monensin treatments, the puncta formation was less efficient and appeared later when compared to Auranofin. None of them behaved as the classical LMP inducer Siramesine, although Thiostrepton and the positive control lapatinib reached a similar level of puncta formation at later time points. Treatment with the autofluorescent drug Quinacrine resulted in a high and steady green fluorescence signal (Fig. 2a). The images of the cells at 12 h time point for Auranofin, Monensin, Niclosamide, Quinacrine and Siramesine treatments are shown in Supplementary Fig. 2a. To test whether the puncta formation for Quinacrine (Supplementary Fig. 2a) was due to the accumulation of Gal3, we treated MCF7 cells with 5 µM Quinacrine for 6 and 24 h, and next fixed (to release the drug from the cells) and stained the cells with an anti-Gal3 antibody to image the appearance of Gal3 puncta (Fig. 2b, Supplementary Fig. 2b).

Cell death was induced within 24 h with a 5 µM concentration of all the drugs that induced LMP during the 24 h treatment (Auranofin, Niclosamide, Monensin, Quinacrine and Siramesine) (Fig. 2c). Colchicine and Podophyllotoxin induced neither LMP, nor induced the same level of cell death as the other drugs (Fig. 2c). Similarly, live cell counting revealed decreasing cell numbers for all the drugs, although for Podophyllotoxin, Colchicine and Thiostrepton the decrease during the first 24 h was less than for the other compounds (Supplementary Fig. 2c). With the 5 µM concentration used, all of the drugs initiated cell death within 48 h treatment (Fig. 2c).

### Titration of the drugs for evaluation of the signalling pathways affected

**Fig. 3:**
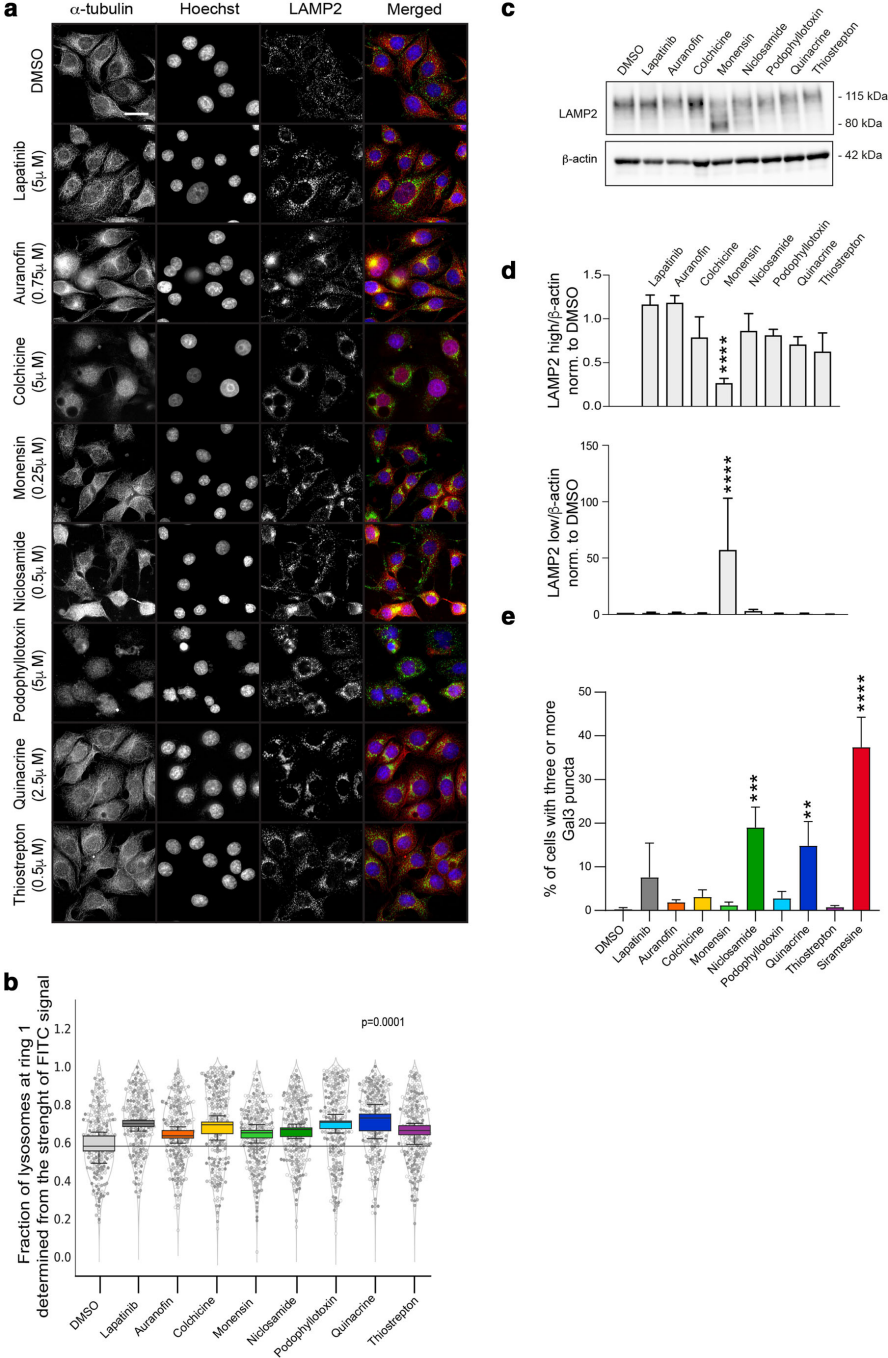
Lysosome distribution, LAMP2 expression and LMP in p95-ErbB2-MCF7 cells treated with the lowest lysosome distribution-affecting drug concentrations. **a** Representative immunofluorescence images of lysosome distribution in p95-ErbB2-MCF7 cells treated for 24 h with vehicle (DMSO), lapatinib (5 µM) and indicated compounds and concentrations. Cells were fixed and stained as in 1a. The images were taken using a ScanR high-throughput microscope and subjected to deconvolution. Scalebar: 20 μm. **b** Quantification of lysosome distribution. Images were acquired as for 1a. Lysosome distribution was scored as the fraction of lysosomes found in the perinuclear ring (~ 4 μm wide) and plotted as a box plot from 5 biological replicates of triplicate experiments. Each circle represents a single cell measurement, grey levels represent separate experiments and the violin blot indicates the distribution of cells. The horizontal line presents the median value of the DMSO control. *P*-values are < 0.0001 (t-test) for each drug compared to DMSO. **c** LAMP2 detection in Western blots of p95-ErbB2-MCF7 cells treated with the titrated drug concentrations (as in 3a) for 24 h. β-actin was used as a loading control. **d** Quantification of the LAMP2 Western blots. The LAMP2 large form (LAMP2 high; upper columns) and the LAMP2 non-glycosylated smaller form (LAMP2 low; lower columns) were normalized to β-actin and finally to DMSO treatment using Image J. Quantification is presented as mean and standard deviation from three independent blots. ANOVA, ****p < 0.0001. **e** Gal3 puncta detection in p95-ErbB2-MCF7 cells treated 24 h as in 3a. Gal3 puncta formation for LMP detection was done after immunostaining with a Gal3 antibody. The images were acquired using an ImageXpress microconfocal system with 60x objective. Analyses were performed using MetaXpress software. Cells with three or more puncta were defined as Gal3 puncta positive. The results are presented as mean and standard deviation of three biological replicates done in triplicate, ANOVA

 Since some drugs were cytotoxic at 5 µM concentrations, we used a viability assay to titrate the drugs to the concentrations that were causing 5 % or less cell death upon 48 h treatment. This was done to identify the lowest concentrations sufficient to change lysosome distribution while inducing a minimal amount of cell death (Supplementary Fig. 3a), in order to use these concentrations to study the signalling pathways and mechanisms affecting lysosome distribution. For this, we also manually evaluated the co-existence of lysosomes in the cellular periphery, so that in the cases where a high accumulation of perinuclear lysosomes coincided with clear LAMP2 staining in invadosome-like structures, higher concentrations were chosen. These lower drug concentrations were used to confirm the results and to study the perinuclear lysosome distribution in more detail. Deconvolution of the images was performed to improve their resolution (Fig. 3a). Next, the perinuclear lysosome distribution was quantified using an additional method, by quantifying the strength of the FITC signal (LAMP2 staining) in the perinuclear ring of a diameter of 4 μm (Fig. 3b). All the drugs were still significantly inducing an accumulation of perinuclear lysosomes (Fig. 3b). We also analysed the LAMP2 levels upon 24 h drug treatment and found them to be steady, except with Monensin (Fig. 3c, d). Using Western blotting (Supplementary file 1) we found that Monensin-treatment resulted in increased expression of a smaller, 80 kD form of LAMP2 that coincided with the decrease of the expression of the 115 kD form of LAMP2, suggesting a Monensin-induced de-glycosylation of LAMP2 (Fig. 3c, d). Because ErbB2 inhibition induces perinuclear lysosome accumulation [21], we used these lower concentrations and 90 min treatments to verify whether some of them could be directly inhibiting ErbB2 downstream signalling (Supplementary Fig. 3b). In lapatinib-treated cells ERK1, ERK2 Thr202/Tyr204 and Akt S473 phosphorylation was inhibited, as expected. Moreover, both lapatinib and Niclosamide inhibited phosphorylation of the mTOR substrate p70S6K. The activity of PAK4, a kinase responsible for the ErbB2-induced phosphorylation of Myeloid Zinc Finger 1 (MZF1) and invasion [6, 27], was not inhibited by any of the treatments, as was evident from the unaltered phosphorylation of the PAK4 substrate cofilin (Supplementary Fig. 3b). We used these low drug concentrations to examine their ability to induce LMP in p95-ErbB2-MCF7 cells and found that only Niclosamide, Quinacrine and the positive control Siramesine could still induce significant amounts of LMP during 24 h treatment (Fig. 3e).

### The effect of drugs on autophagy and cathepsin B

**Fig. 4:**
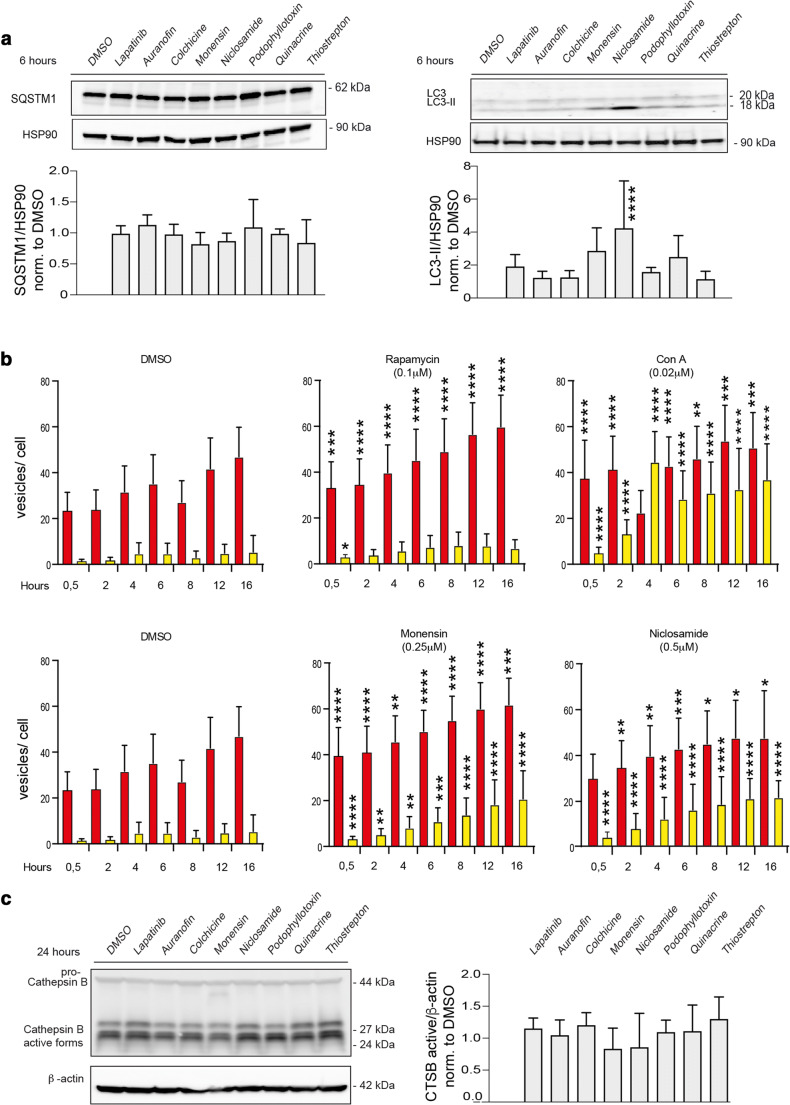
Drug effect on autophagy and cathepsin B expression. **a** Western blots of autophagic markers SQSTM1 (left side blots) and LC3-II (right side blots) after 6 h treatment of p95-ErbB2-MCF7 cells with the lowest efficient concentrations of the indicated drugs. Corresponding quantifications presented as mean and standard deviations from 3–4 independent experiments are shown under the blots. HSP90 was used as a loading control. Statistics were calculated with ANOVA. **b** Autophagic flux measurements of drug-treated tfLC3-MCF7 cells. Cells were treated with the lowest efficient concentrations of the indicated drugs. Two nM ConA and 0.1 µM Rapamycin were used as autophagy controls. The images were acquired at 0,5 h to 16 h after treatment using an ImageXpress microconfocal system with 40x objective. Analyses were performed using MetaXpress software. Red columns represent autophagolysosomes and yellow columns represent autophagosomes. The mean values represent means of three biological replicates done in triplicate wells. Statistics were calculated with ANOVA. (**c**) Western blot of cathepsin B after 24 h treatment of p95-ErbB2-MCF7 cells. β-actin was used as a loading control. Quantifications represent means and standard deviations of three independent experiments

To next test whether drugs can induce early autophagy, we treated the p95-ErbB2-MCF7 cells for 6 h with titrated concentrations of drugs to detect changes in the sequestrosome (SQSTM1, p62) levels, which would indicate major changes in bulk autophagy. The level of SQSTM1 was found to be stable (Fig. 4a, left side blots), suggesting that putative autophagic changes were minor. We subsequently assayed autophagy via detecting LC3 lipidation (Fig. 4a, right side blots). For both Monensin and Niclosamide, a slight accumulation of the 18 kD form of LC3 corresponding to lipidated LC3 (LC3-II) was observed (Fig. 4a, right side blots). A 90 min Niclosamide treatment resulted in inhibition of mTOR signalling, an event that is known to induce autophagy (Supplementary Fig. 3b). An autophagy flux assay was used to evaluate whether these drugs can affect autophagosome maturation using cells that are stably overexpressing the tandem fluorescent autophagy indicator mRFP-GFP-LC3 (tfLC3-MCF7 cells) [24, 28] (Fig. 4b). The subcellular localization of mRFP-GFP-LC3 is autophagy-dependent and upon autophagosome formation, mRFP-GFP-LC3 is translocated to the autophagosomal membranes via lipidated LC3, inducing the appearance of yellow autophagosome vesicles. During later steps of autophagy, when the autophagosomes fuse with lysosomes, LC3 is located to the inner side of autolysosome and the GFP signal is quenched due to the acidic pH of the autolysosome, whereas RFP remains stable, making autolysosomes appear as red, puncta-like structures (Fig. 4b, red columns) [25, 28]. Induction of autophagy is visualized by increases in yellow and red puncta, whereas autophagy inhibition is seen as an increase in yellow puncta, often concomitant with a decrease in the number of red puncta. When comparing DMSO treatment with Rapamycin or Concanavalin A (ConA) (positive controls), and with Monensin and Niclosamide, the Monensin and Niclosamide treatments started with a higher number of red LC3 puncta as did both positive controls, the numbers of which increased as a function of time (Fig. 4b). The increase in yellow puncta in Monensin and Niclosamide treated cells was statistically significant and resembled ConA treatment more than that of Rapamycin (Fig. 4b, Supplementary Fig. 4). Western blot analysis was used to detect cathepsin B expression upon 24 h treatment to assess whether the compounds interfere with the maturation and activation of this central lysosomal protease needed for the execution of lysosome-mediated cell death. The expression of the active forms of cathepsin B were found to be steady (Fig. 4c).

### Effects of drugs on the invasion of lapatinib‐resistant breast cancer spheroids and ovarian cancer organoids

**Fig. 5:**
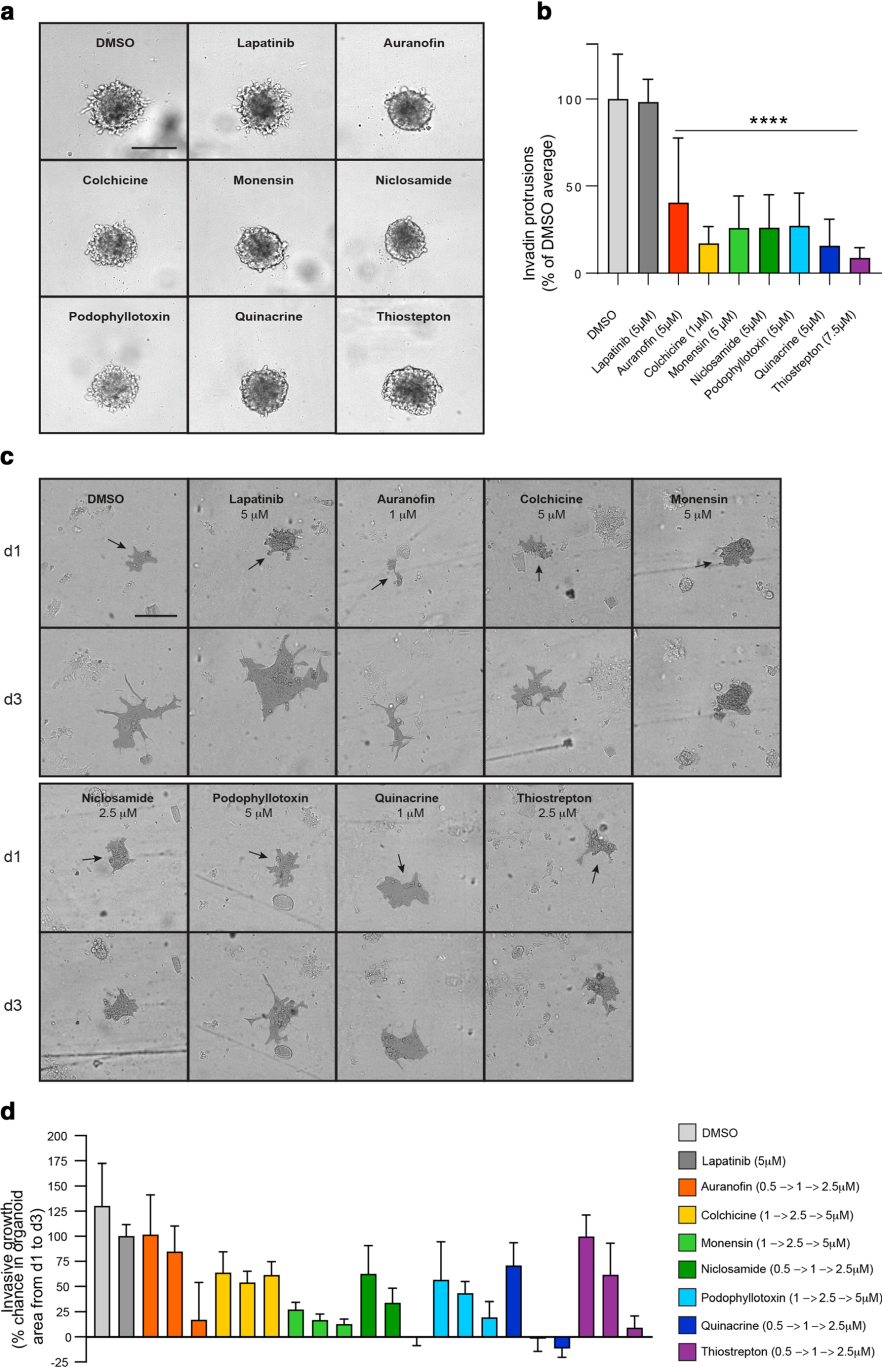
Assessment of drug efficiencies in 3D invasion assays using lapatinib-resistant MT2 (LR-MT2) breast cancer cell spheroids and lapatinib-resistant OVC316 ovarian cancer organoids. **a** LR-MT2 cell spheroid invasion in 3D cultures in BME matrix. Spheroid cultures were treated with the indicated drugs upon their establishment. Images were taken at 48 h using ImageXpress. Scale bar: 200 μm. The drug concentrations were titrated to the lowest effective concentrations when inhibition of invasion of the spheroids was evaluated and the images shown are representatives of most efficient treatments of spheroids prepared in triplicates from three biological replicates. **b** Quantification of the invasive growth of LR-MT2 3D spheroids based on the number of invading protrusions and including images from 5a. The mean represents a mean of three biological replicates done in triplicate wells. Statistics were calculated with ANOVA. **c** OVC316 organoid invasion in BME matrix. Organoids were isolated from OVC316 tumors and embedded in BME matrix and treated with three different concentrations of the indicated drugs. Organoids were imaged at days 1 (d1) and 3 (d3) of their establishment using ImageXpress with 10x objective. Analyses were performed using MetaXpress software. Scale bar: 200 μm. The images are representatives of organoids treated with three different concentrations of the indicated drugs and the selected concentrations are indicated in the images. One representative organoid for each treatment is highlighted. **d** Quantification of invasive growth of OVC316 organoids from 5c. Analyses were performed using MetaXpress software. Organoids were prepared and treated with three different drug concentrations in duplicates and as two technical repeats

HER2 positive breast cancer cells are generally much less aggressive *in vitro* and in mouse xenograft models than in patients. We chose the following two model systems for invasion studies because (a) they are resistant to 2nd line HER2 inhibitor treatment, (b) they have retained the typical aggressive invasiveness of HER2 positive cancer and (c) they can grow as 3-dimensional structures, thus better representing actual tumor growth than can be achieved with 2-dimensional cultures. To test the effect of the drugs on invasiveness, we used a mammary cancer cell line, MT2, expressing full-length Neu (rodent ErbB2), isolated from a murine mammary tumor lung metastasis [29]. We grew the cells in the presence of lapatinib for several months until we found that the cells had acquired lapatinib-resistance. The resulting cell line was called LR-MT2. In a LR-MT2 3D spheroid invasion assay, most of the drugs were found to be more efficient in inhibiting the formation of invasive cellular protrusions than lapatinib (Fig. 5a, b). We additionally tested the drugs *ex vivo* using a highly invasive ErbB2-positive high-grade human serous ovarian cancer cell line OVC316 that expresses full-length ErbB2 [30]. We isolated OVC316 xenograft tumors to prepare organoids and embedded them in BME matrix to follow their invasive growth and response to the respective drug treatments (Fig. 5c, d). All the drugs were found to be more efficient in inhibiting organoid invasion than lapatinib. The organoids were resistant to lapatinib treatment, and their invasive growth upon lapatinib treatment did not differ markedly from that of those treated with the vehicle control DMSO (Fig. 5c, d).

## Discussion

Pericellular lysosomes are important mediators of cancer cell invasion due to their ability to induce and increase extracellular matrix degradation. This function is especially driven by oncogene-induced, increased expression, activity and secretion of lysosomal cysteine cathepsins B and L [6, 8]. Several drugs inhibiting these cathepsins have been developed as invasion inhibitors, but none of them made it to the clinic mainly due to toxicity problems [31]. Here we took a different approach by identifying commonly used drugs that can direct lysosomes to the perinuclear area, away from the plasma membrane, physically preventing them to secrete their digestive contents into the extracellular space. Quantification of the invasion capacity indicated that all seven identified drugs inhibited the invasive growth of two 3-dimensional cellular model systems of 2nd line HER2 inhibitor lapatinib resistant cancers. Most efficient against lapatinib resistant LR-MT2 mammary cancer spheroids was Colchicine, already at 1 µM concentration. The most efficient drugs against the invasive growth of OVC316 ovarian cancer organoids were Niclosamide, Quinacrine and Thiostrepton. Even though slight differences were noted between the two model systems with respect to which of the drugs was most potent, they all efficiently inhibited invasive growth in the 2nd line HER2-inhibitior resistant model systems. The differences in the effects of the drug responses between the model systems could be caused by different signalling pathways activated and/or genetic differences between the systems. They may thus support a possible scenario by which in the future a set of potentially efficient lysosome targeting drugs could be tested against tumors in a personalized manner, using tumor tissue obtained from patients to identify the most efficient drug in each case. Moreover, our initial goal to find drugs that could prevent invasion of HER2 inhibition resistant cancer models while retaining most of the activity of cathepsin B and thus the cell’s ability to undergo lysosome-dependent cell death was achieved. The seven compounds identified are very divergent in their structure. Only one of them, Niclosamide, had an inhibitory effect on the activity of a direct ErbB2/HER2 downstream signalling kinase (mTOR), whereas the others were targeting invasion downstream of ErbB2 effector kinases, supporting their usability in ErbB2 inhibition-resistant cases (Table 1).

**Table 1:**
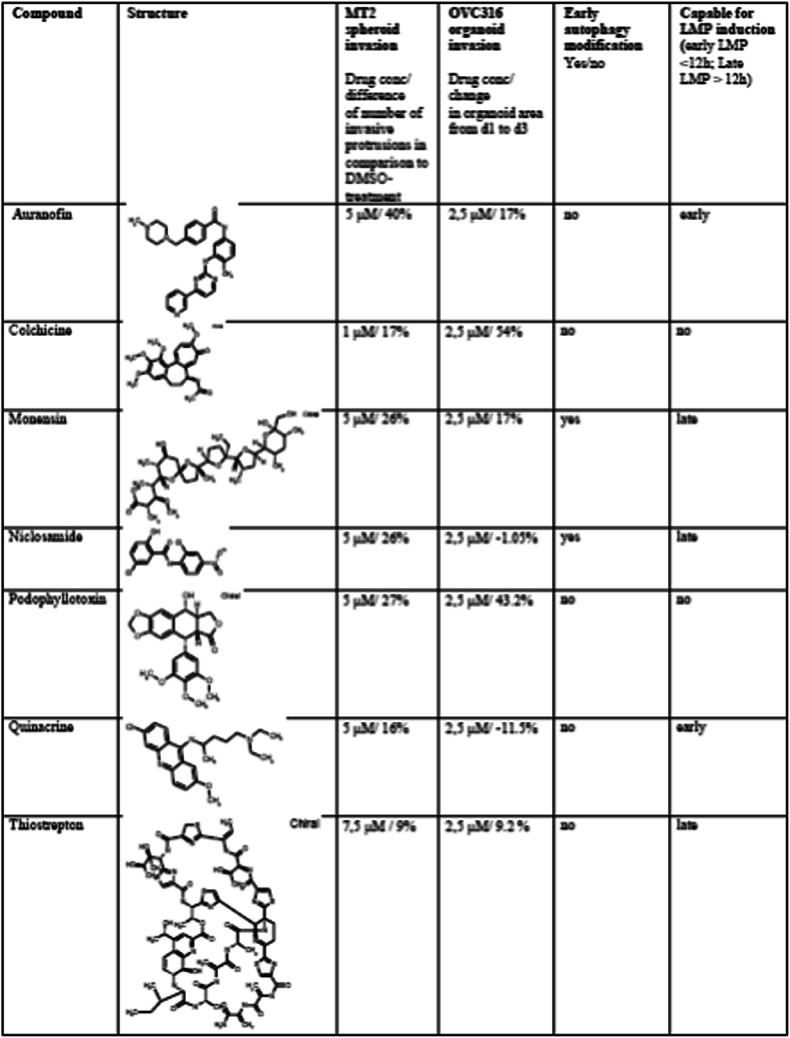
Seven compounds and their effects on invasion, autophagy modification and lysosomal membrane permeabilization

For MT2 spheroid invasion evaluation, the most efficient invasion inhibiting concentrations were used for each compound. For the evaluation of OVC316 tumor organoid invasion, 2,5 µM drug concentrations were used. The ability to induce LMP was evaluated as “early” if Gal3 puncta formation (3 or more puncta per cell) was evident before 12 h and “late” if it became evident after 12 h.

When comparing their ability to induce LMP and to modulate autophagy, we found out that the drugs could be roughly divided into four groups, i.e., those (1) inducing early LMP (LMP puncta detection by 12 h; Auranofin and Quinacrine), (2) inducing early autophagy with late LMP (LMP by 48 h; Monensin and Niclosamide), (3) not inducing autophagy with late LMP (Thiostrepton and lapatinib) and (4) not inducing autophagy nor LMP (Colchicine and Podophyllotoxin). Only Monensin and Niclosamide treatments affected autophagy. While the increase in the red LC3 puncta formation induced by these drugs resembled that of Rapamycin, the increase in the yellow puncta formation, especially at later timepoints, resembled that of ConA. Since Rapamycin is known to induce autophagy, and ConA treatment often leads to inhibition of autophagy, our results suggest that Monensin and Niclosamide treatment initially induced autophagy, perhaps as a survival mechanism, but led to its inhibition and cell death at later timepoints. Both Monensin and Niclosamide are known to increase lysosomal pH [32, 33], which could lead to autophagy inhibition. Interestingly, we identified lapatinib as a novel LMP-inducing drug in lapatinib sensitive p95-ErbB2-MCF7 cells, suggesting that lapatinib can induce or sensitize cells to lysosome-dependent death, raising the possibility that lapatinib may serve as a CAD.

Niclosamide and Quinacrine were identified as strong LMP inducers in p95-ErbB2-MCF7 cells even at low concentrations. Generally, we found that the drugs that induced LMP were the most efficient inhibitors of invasive growth of spheroids and organoids, the only exception being Colchicine, which was one of the most efficient inhibitors of invasive growth without inducing LMP. In case of Auranofin, cell death was evident already several hours before the induction of LMP, suggesting that Auranofin can induce different types of cell death. Monensin blocks the formation of complex oligosaccharides [34], which is a likely reason for the observed de-glycosylated, smaller size LAMP2 upon 24 h Monensin-treatment. De-glycosylation of LAMP2 should make lysosomal membranes more sensitive for permeabilization (LMP) and lysosomal cell death [8]. This is not what we observed. Instead, we found that an over 10-fold higher Monensin concentration was needed to induce LMP at later timepoints. Monensin treated cells likely compensated the lack of lysosome membrane protective LAMP2 glycosylation to retain the integrity of their lysosomal membranes. Colchicine and Podophyllotoxin induced neither LMP, nor the same level of cell death than the other drugs, despite the relatively high concentrations used (5 µM), indicating that their mechanism of action was different from the others. Interestingly, their perinuclear lysosome accumulation patterns also differed from those achieved by the other drugs (Fig. 3b), which was also apparent from immunofluorescent images.

 All the seven drugs are known, or have recently been identified as efficient anti-viral, anti-microbial or anti-inflammatory agents. Auranofin, a rheumatoid arthritis drug functions partially by preventing cellular release of lysosomal enzymes [35]. It can efficiently target SARS-CoV-2 replication in human cells killing 95 % of the virus within 48 h at a 4 µM concentration [36]. Another rheumatoid arthritis drug, Colchicine, is an anti-inflammatory drug that is being tested in clinical trials as a potential inhibitor of SARS-CoV-2-induced overreactions of the immune system, i.e., so-called cytokine storm. Monensin is an ionophore and an antibiotic that is used to prevent infections in the intestinal mucosa. It is an inhibitor of endosomal acidification and inhibits the cellular entry of semliki forest, rabies, vesicular stomatitis, influenza H5N1 and Ebola viruses [37]. Niclosamide exhibits a similar activity and, additionally, against human immunodeficiency virus type 1, herpes simplex virus 1, vaccinia virus [37], SARS-CoV [38] and SARS-CoV-2 [39]. Both drugs have a preference to inhibit virus entry at the pre-fusion stage in addition to inhibiting viral replication [40]. Similarly, anti-viral, anti-bacterial and anti-inflammatory activity has been attributed to Podophyllotoxin, Quinacrine and Thiostrepton. Our work suggests that the anti-viral activities of these drugs could be connected with their ability to target lysosomes at the cellular level. For example, cellular entry of coronavirus requires lysosomes, since it demands proteolytic processing of their surface spike proteins in lysosomes, especially by cathepsin B [41, 42]. The coronavirus entry mechanism relies on endosomes as their main transporters to lysosomes. Based on our work, it would be interesting to investigate if for example drug-induced increased perinuclear lysosome positioning can affect endosome-mediated viral entry.

Lysosomal contribution to platinum as well as multidrug resistance is expected to occur via altered lysosomal functions and deregulated autophagy [43, 44]. Thus, targeting lysosomes may ameliorate the treatment of these, often untreatable cancers. Auranofin has shown to be efficient against triple negative breast cancer cells and xenografts [45] and it is currently tested in a clinical trial as a combinatory treatment with sirolimus/rapamycin against treatment resistant ovarian cancer (https://clinicaltrials.gov/ct2/show/ NCT03456700). The fact that Colchicine can interfere with cell division by disrupting microtubule assembly and disassembly during mitosis, and that an arsenal of anti-cancer drugs already exists that act via microtubule assembly inhibition, may explain why it has not been taken into clinical trials. However, considering its potential multi-purposing value as an potential anti-inflammatory/anti-SARS-CoV-2 affecting agent and as an inhibitor of lysosome-mediated invasion, its clinical use might be reconsidered. Monensin can induce apoptosis especially in prostate cancer cells undergoing epithelial-mesenchymal transition [46], whereas Niclosamide can direct pericellular lysosomes of DU145 prostate cancer cells to perinuclear positions, indicating its effect on lysosome-mediated invasion [47]. The commonly used anticancer drugs etoposide and teniposide are derivatives of Podophyllotoxin. They are inhibitors of tubulin assembly and activators of apoptosis and thus capable of affecting microtubule dynamics and, thereby, lysosome distribution. Recently, it was suggested that Quinacrine could be repurposed against treatment-refractory cancers including breast and ovarian cancers, although its mechanism of action has not been fully understood [48]. We identified Quinacrine as an efficient inducer of lysosome membrane permeabilization, a phenomenon that could be involved in its ability to sensitize cancer cells to platinum-based compounds. Thiostrepton may be combined with chemotherapy targeting breast and ovarian cancers through its ability to inhibit the expression of the cancer stemness regulator FOXM1, which leads to attenuation of stemness [49] and chemosensitivity [50], respectively.

## Conclusions

Here we describe the identification and partial characterization of seven structurally very divergent drugs as efficient inhibitors of invasive growth of lapatinib-resistant HER2 positive breast cancer spheroids and HGSOC ovarian cancer organoids, all functioning via lysosomes. We classify them into four groups based on their effect on LMP and autophagy modification. Six of these drugs have previously been identified as efficient antiviral agents, and the fact that four of them are efficient against SARS-CoV-2 in cellular studies implicates that some of them may become extra attractive for being tested in clinical trials. Our work suggests that targeting lysosomes may be an efficient way to combat treatment resistant, invasive HER2 positive cancer cells. The possibility that each of the seven drugs can be efficient against this type of cancer, which is generally difficult to treat, may further increase their attractiveness for testing in clinical trials.

## Supplementary Information

ESM 1 (PDF 1.24 MB)

ESM 2 (XLSX 671 KB)

ESM 3 (PDF 5.06 MB)

ESM 4 (PDF 4.07 MB)

## Data Availability

Data and material are available upon reasonable request.

## Abbreviations

LMP: lysosomal membrane permeabilization
HGSOC: high-grade serous ovarian cancer
LAMP2: lysosomal membrane protein 2
TDM1: trastuzumab emtansin
CAD: cationic amphibilic drug
FITC: fluorescein isothiocyantae
DMSO: dimethylsulfoxide
PNLA: perinuclear lysosome accumulation score
Gal4: galectin 4
EGFP: enhanced green fluorescent protein
MZF1: myeloid zinc-finger 1
PAK4: p21-activated kinase 4
ERK1/2: extracellularly regulated kinase 1/2
mTOR: mammalian target of rapamycin
SQSTM: sequestrosome
ConA: concanavalinA
LC-3: microtubule-associated protein 1A/1B-light chain 3
