# Supplementary material for: Identification of lysosome‐targeting drugs with anti‐inflammatory activity as potential invasion inhibitors of treatment resistant HER2 positive cancers
Source: Cell Oncol (Dordr). 2021 May 3;44(4):805–20. doi: 10.1007/s13402-021-00603-2 (PMC8090911; doi:10.1007/s13402-021-00603-2)
Supplement: Supplementary file 1 — (PDF 1.24 MB) [file 13402_2021_603_MOESM1_ESM.pdf]

**Supplementary Information,** Bredahl Hansen et al. CEON-D-20-00795

## Supplementary Materials

Primary antibodies

| Antigen | Application | Host species | Dilution | Company/source | Cat# |
|---|---|---|---|---|---|
| $\alpha$-tubulin | IF | pAb Rabbit | 1:1000 | Abcam | ab15246 |
| Akt (p-S473) | WB | mAb Rabbit | 1:500 | Cell Signaling | 4060 |
| Akt (p-T308) | WB | pAb Rabbit | 1:2000 | Cell Signaling | 9275 |
| Akt (Pan) | WB | mAb Mouse | 1:1000 | Cell Signaling | 2920 |
| $\beta$-actin | WB | mAb | 1:1000 | Sigma-Aldrich | A2228 |
| Cathepsin B | WB | mAb Mouse | 1:1000 | Dr. Ekkehard Weber | 3E4 + 6D5 |
| Cathepsin L | WB | mAb Mouse | 1:1000 | Dr. Ekkehard Weber | AB-33/1 |
| CIP2A | WB | pAb Rabbit | 1:500 | Bethyl Laboratories | A301-454A |
| Cofilin | WB | pAb Rabbit | 1:300 | Cell Signaling | 3318 |
| ErbB2 | WB | mAb Mouse | 1:600 | Thermo Fisher | MS-730-P0-A |
| ERK2 | WB | pAb Goat | 1:1000 | Santa Cruz | sc-154-G |
| Galectin 3 | IF | mAb Rat | 1:200 | Sigma-Aldrich | MABT51 |
| GAPDH | WB | mAb Mouse | 1:2000 | Abcam | Ab189095 |
| HSC70 | WB | mAb Mouse | 1:1000 | Prof. Boris Margulis | N69a |
| HSP90 | WB | mAb Mouse | 1:1000 | Santa Cruz Biotech | sc-13119 |
| LAMP2 | IF | mAb Mouse | 1:500 | DSHB(*) | H4B4c |
| LC3B | WB | mAb Rabbit | 1:500 | Cell Signaling | 3868 |
| MZF1 | WB | pAb Rabbit | 1:500 | Abcam | ab64866 |
| MZF1-pS27 | WB | mAb Rabbit | 1:500 | Brix et al. 2019 | NA |
| p-Cofilin | WB | pAb Rabbit | 1:2000 | Cell Signaling | 3311 |
| p-ErbB2 | WB | mAb Rabbit | 1:1000 | Cell Signaling | 2243 |
| p-ERK1/2 | WB | mAb Rabbit | 1:2000 | Cell Signaling | 4376 |
| p-p70S6k | WB | mAb Mouse | 1:2000 | Cell Signaling | 9206 |
| p-PAK4/5/6 | WB | pAb Rabbit | 1:1000 | Cell Signaling | 3241 |
| P62 (SQSTM1) | WB | pAb Rabbit | 1:1000 | Enzo Lifescience | BML-PW9860 |
| PAK4 | WB | pAb Rabbit | 1:1000 | Cell Signaling | 3242 |
| ps70S6k | WB | pAb Rabbit | 1:2000 | Cell Signaling | 9202 |
| ULK1 | WB | mAb Rabbit | 1:2000 | Cell Signaling | 8054 |

(*) Developmental Studies Hybridoma Bank.

Secondary antibodies

| Antigen | Alexa Fluor | Dilution | Company | Cat# |
|---|---|---|---|---|
| Donkey anti-Mouse | 488 | 1:1000 | Thermo Fisher | A-21202 |
| Donkey anti-Mouse | 568 | 1:1000 | Thermo Fisher | A-10037 |
| Donkey anti-Rabbit | 594 | 1:1000 | Thermo Fisher | A-21207 |
| Goat anti-Rat | 488 | 1:1000 | Thermo Fisher | A-11006 |

Drugs

| Product | Company | Cat# |
|---|---|---|
| Auranofin | Sigma-Aldrich | A6733 |
| Colchicine | Sigma-Aldrich | C9754 |
| Monensin | Sigma-Aldrich | M5273 |
| Niclosamide | Bionordika / Medinova | CC-10649 |
| Podophyllotoxin | Sigma-Aldrich | PP4405 |
| Quinacrine | Sigma-Aldrich | CDS020709 |
| Thiostrepton | MERCK Millipore | 598226 |
| ConA | Sigma-Aldrich | 27689 |
| Digitonin | Sigma-Aldrich | D141 |
| Lapatinib Ditosylate | Santa Cruz | SC-202205 |
| Rapamycin | Sigma-Aldrich | R0395 |
| Siramesine | Cayman Chemical | 21817 |

Cell culture / Chemicals / Kits

| Product | Company | Cat# |
|---|---|---|
| 4-Methylumbelliferyl-N-Acetyl-B-D-Glucos (NAG substrate) | VWR | 474502 |
| 10xMEM | Sigma | D2429 |
| BSA | VWR | 422361V |
| Clarity™ Western ECL Substrate | BioRad | 170-5061 |
| cOmplete™ Mini Protease Inhibitor Cocktail | Roche | 4693124001 |
| Criterion™ TGX™ 18-well gel | BioRad | 5671084 |
| Criterion™ TGX™ 26-well gel | BioRad | 5671085 |
| Cultrex® GFR Basement Membrane Extract, Type 2 | Amsbio | 3533-010-02 |
| DMEM + GlutaMAX™-I | Gibco | 31966-021 |
| DMEM/F12 | Gibco | 11039-021 |
| DMSO | VWR | WN182 |
| DPBS | Gibco | 14190-094 |
| FBS | Gibco | 10270-106 |
| Hoechst 33258 | Sigma-Aldrich | B2883 |
| Hoehst 33346 | Sigma-Aldrich | B2261 |
| Insulin | Sigma-Aldrich | I9278 |
| LCS1-Violet | AAT Bioquest | 17543 |
| Novex™ Sharp Pre-stained Protein Standard | Invitrogen | LC5800 |
| Pen Strep | Gibco | 15140-122 |
| PFA 37% | VWR | |
| PhosSTOP | Roche | 4906845001 |
| Ponceau S solution | Sigma-Aldrich | P7170 |
| Propidium iodide solution | Sigma-Aldrich | P4864 |
| RPMI 1640 + GlutaMAX™-I | Gibco | 61870-010 |
| Sir-tubulin | Spirochrome | SC002 |
| Skim Milk Powder | Sigma-Aldrich | 70166 |

| | | |
|---|---|---|
| Sodium Bicarbonate Solution | Gibco | 25080-060 |
| Triton X-100 | Sigma-Aldrich | T9284 |
| Trypsin | Gibco | 12604-013 |
| Tween 20 | Sigma | 274348 |

Plastics

| Product | Company | Cat# |
|---|---|---|
| 4titude | 4titude | 221 |
| Corning® 96-Well Half-Area Microplate | Sigma-Aldrich | 3694 |
| Corning® Costar® Ultra-Low Attachment Round Bottom 96-Well Plate | Sigma-Aldrich | 7007 |
| Greiner CELLSTAR® 96-well plates | Sigma-Aldrich | 655090 |
| Nunc™ 6-Well Cell-Culture plate | Thermo Fisher Scientific | 140675 |
| Nunc™ 96-Well Cell-Culture plate | Thermo Fisher Scientific | 167008 |
| NuncTM Cell Culture Treated Flask, 175 $cm^2$ | Thermo Fisher Scientific | 178883 |
| NuncTM Cell Culture Treated Flask, 80 $cm^2$ | Thermo Fisher Scientific | 178905 |

Instruments

| Machine | Company |
|---|---|
| Bioruptor™ | Diagenode |
| Celigo Image Cytometer | Nexcelom Bioscience |
| ImageXpress® Micro Confocal | Molecular Devices |
| IncuCyte ZOOM® Live-Cell Analysis System | Sartorius |
| LAS-4000 | Fujitsu Life Science |
| LSM700 | Zeiss |
| SpectraMax® iD3 Multi-Mode Microplate Reader | Molecular Devices |
| Trans-Blot® Turbo™ Transfer System | BioRad |

## Supplementary methods

### Tissue Culture

MCF7 M8-tTAS-pTRE-ΔNErbB2 cells (in the text referred to as p95-ErbB2-MCF7 cells) and the corresponding vector cells were cultured, and induced as described elsewhere [1]. Induced cells were cultured in RPMI1640 + GlutaMAX™-I (GIBCO) medium supplemented with 6% fetal bovine serum (FBS; GIBCO) and 0,25% penicillin and streptomycin (P/S; Sigma-Aldrich)

as were the MCF7-pEGFP-Galectin 3 and tfLC3-MCF7 reporter cell lines. The experiments with p95-ErbB2-MCF7 cells were carried out at passage three to five after induction of the expression of the p95 ErbB2. SKOV3ip1 cells were cultured as described previously [2]. Lapatinib-resistant MT2 (LR-MT2) cells were cultured in DMEM/F-12 medium (GIBCO) supplemented with 10% FBS, 0,25% P/S, 10 ng/ml EGF (Sigma-Aldrich), 1 μg/ml hydrocortisone (Sigma Aldrich) and 5μg/ml insulin (Sigma-Aldrich). To ensure maintenance of the resistant phenotype, 10μM lapatinib (Santa Cruz) was added to the culture medium for 5 days post thawing. The cells were cultured in lapatinib-free medium for at least 5 days prior to experiments. OVC316 ovarian cancer cells [3] were grown in DMEM/MEGM (GIBCO/Lonza) supplemented with 5% FBS, 0.25% P/S and 5 μg/ml insulin.

Immunocytochemistry

Cells were seeded in black 96-well plates with clear bottoms (4titude) 24-48 h prior to treatment and fixed 24 h post treatment. For fixation, preheated (37°C) paraformaldehyde (PFA; VWR) was added to the cells for a final concentration of 4%. Cells were washed twice with PBS, incubated with cold methanol (Sigma) for 3 min at -20°C and washed twice with PBS. Blocking buffer (PBS with 1% bovine serum albumin (BSA) (Sigma), 0.3% Triton X-100 (Sigma) and 5% FBS) was added for 30 min at room temperature. Primary antibodies were diluted in blocking buffer and incubated overnight at 4°C. Cells were washed twice with washing buffer (PBS with 1% BSA, 0.1% Triton X-100) and incubated for one hour at room temperature with secondary antibodies diluted in washing buffer supplemented with 5% FBS. Cells were then washed three times for 5 min in PBS with 0.05% Tween (Sigma), 5 min in PBS with 50 μg/ml Hoechst-33258 (Sigma) and two times 5 min in PBS. Finally, cells were left in 200 μl of MilliQ H2O. Images were acquired using the ImageXpress® Micro Confocal System and analyzed using the MetaXpress® High-Content Image Acquisition and Analysis Software.

Cell viability and cell death measurements

For cell viability assay, cells were seeded in 96-well plates 24-48 h prior to treatment (Greiner; Sigma Aldrich). Following 24h of treatment, cells were stained for 10 min at 37°C, in the dark, with 80 μg/ml Hoechst-33342 (Sigma-Aldrich) and 0.55 μg/ml propidium iodide (Sigma-Aldrich) solution in complete growth media, to determine the total cell count and the count of dead cells, respectively. Cell death count and total count were determined using the Celigo Image Cytometer (Nexcelom Bioscience) and analysed using the Celigo Software using following excitation and emission wavelengths: Hoechst-33342: Excitation 350, Emission 461.

Propidium iodide: Excitation 535, Emission 617. Dead cell detection with ImageXpress images (Fig 2) acquired with 40X objective was done firstly by detecting high intensity nuclear stain signal (LCS1-Violet, AAT Bioquest) and secondly by using transmitted light (TL) images exploiting the fact that rounding up cells have more contrast in the TL images and this was enhanced and tresholded to detect them in various steps. When either of the phenotypes was detected, cells were defined as dead. Data on all individual cells detected in MetaXpress, were exported (cell measurements). Subsequent analysis was done in R. Outlier cells (sites with below 5 or above 80 cells) were removed from the dataset. The percentage of dead or live cells was calculated from the total amount of cells detected in each site. Subsequently, the mean of all sites was found for each well. The mean of the three technical replicates was calculated for each drug.

Immunoblotting

Cells were washed twice in PBS and lysed on ice in 2x Laemmli Sample Buffer (LSB) (MilliQ $H_2O$ with 125 mM Tris, 20% glycerol, 140 mM sodium dodecyl sulfate (SDS), 0.2 μg/ml bromophenol blue) supplemented with protease inhibitors (Complete Mini; Roche), phosphatase inhibitors (PhosSTOP; Roche), 20 μM SUMOylation inhibitor (NEM; Sigma-Aldrich) and 100 μM DL-1,4-Dithiothreitol (DTT; VWR). Lysates were boiled for 10 min, sonicated on a Bioruptor$^{TM}$ (Diagenode) and then separated by SDS-PAGE using precast 4-15% gradient gels (Bio Rad). Novex$^{TM}$ Sharp Pre-stained Protein Standard (Invitrogen) was used for molecular weight estimation. Gels were transferred to a nitrocellulose membrane using the Trans-Blot® Turbo$^{TM}$ Transfer System (Bio Rad) and blocked in Tris-buffered saline, 0.1% Tween 20 (TBST) supplemented with 5% milk. Primary antibodies were diluted in TBST supplemented with 5% BSA (Sigma-Aldrich) and incubated overnight at 4°C on a rocking device. HRP conjugated secondary antibodies were diluted in TBST supplemented with 5% milk (Sigma-Aldrich) and incubated for 1 h at room temperature. The immunoblots were developed using Clarity$^{TM}$ ECL Substrate (Bio Rad), and pictures were acquired using the LAS-4000 (Fujitsu Life Science).

Lysosomal Membrane Permeabilization (LMP)

The LMP assay using eGFP-Gal3-MCF7 cells, has previously been described elsewhere [4]. Four thousand cells / 100 μl / well were seeded into black 96-well cell culture plates with clear bottom. Cells were left for 24-36 h to reach 60-70% confluency. Live cell imaging was

performed using ImageXpress in widefield mode using a 40x air objective. Three biological replicate experiments with three technical replicates were done. Nuclear violet stain was used to detect DNA. LMP assay for autofluorescent drug as well as for p95-ErbB2-MCF7 cells was done from fixed cells that were stained with Gal3 antibody and Hoechst as a nuclear stain. Image analysis in all LMP assays was performed by building a custom module analysis pipeline in MetaXpress. Cell segmentation was done using nuclear stain signal and galectin stain signal in "Cell Scoring Objects" module. Border objects were removed. Data on all individual cells detected in MetaXpress, were exported (cell measurements). Subsequent analysis was done in R. Outlier cells (sites with below 5 or above 80 cells) were removed from the dataset. Cells with above 100 Gal-3 puncta detected were removed, as these were likely caused by poor Gal-3 signal separation. Cells were considered Gal-3 puncta positive if 3 or above puncta were detected. The percentage of live cells that were Gal-3 puncta positive was calculated for each site using information obtained from the cell death measurement. The mean Gal-3 puncta per live cell was calculated per site. The percentage of dead or live cells was calculated from the total amount of cells detected in each site. Subsequently, the mean of all sites was found for each well. The mean of the three technical replicates was calculated for each drug.

Tandem fluorescent autophagy assay

The autophagy assay using the tandem fluorescent LC3 expressing plasmid, tfLC3-MCF7, is described elsewhere [5, 6]. Eight thousand cells / 100 µl / well were seeded into black 96-well cell culture plates with clear bottom. Cells were left for 24-36 h to reach 60-70% confluency. Nuclear violet (0.1 µM) and Sir-tubulin (0.2 µM) (Spirochrome) were added along with drug treatments, to visualize the nuclei and tubulin, respectively. Cells were immediate monitored following drug treatments every two hours, using the widefield mode and 40x air objective on ImageXpress. Nuclear violet was images with the DAPI filter (Ex. 377/54nm, Em. 447/60), Sir-tubulin with the Cy5 filter (Ex. 631/28nm, Em. 692/40nm), mRFP puncta with the Texas Red filter (Ex. 560/32nm, Em. 624/40nm), and GFP puncta was imaged using the FITC filter (Ex. 475/34 and Em. 536/40). Images were subjected to segmentation using the MetaXpress analysis software. Nuclei were identified using as the "Find Round Objects" module and masked using an appropriate threshold from the DAPI filter images and defined as "Nuclei". Whole cells were then identified based on Sir-tubulin in the CY5 filter using an appropriate threshold and defined as "Cells". Border objects were removed. "Nuclei" was subtracted from "Cell", leaving the cytoplasm masked, defined as "Cytoplasm". mRFP and GPF puncta was identified in their respective channels using the "Top Hat" module followed by the "Find

Round Objects" module and defined as "Red Puncta" and "Green Puncta". An overlay was made by "Cytoplasm", "Red Puncta" and "Green Puncta". Puncta that had an overlay consisting of both "Red Puncta" and "Green Puncta" was defined as "Yellow Puncta", and puncta consisting of only "Red Puncta" was defined as "Red only Puncta". The number of the respective puncta was calculated per cell. "Yellow Puncta" being Autophagosomes and "Red only Puncta" being Autolysosomes.

3D spheroid invasion assay

The 3D spheroid invasion assay was done by closely following previously established protocol [7]. Two thousand cells were seeded into 100 µl of each well of a low attachment round-bottom 96-well plate (Corning), spun down at 2500 *g* for 10 min and left to form spheres overnight. To ensure absence of single cells in the wells, the plate was spun down for 5 min at 300 *g* the day after. 75 µl was removed from each well and 25 µl of gel-mix was added (50% Cultrex® RGF BME, 37.4% DMEM, 7.4% 10xMEM, 3,7% Sodium Bicarbonate Solution and 1.5% FBS) and left to solidify for 20 min at 37°C. Finally, treatment was added in FBS enriched media (12% FBS) and invasion was imaged with a 10x objective at 24 h and 72 h using the ImageXpress® Micro Confocal System.

Olympus ScanR imaging

Cells were imaged with 40x objective using the standard DAPI (for the Hoechst signal) and FITC (for LAMP2) filters. Image analysis was performed with the ScanR Analysis software. Nuclei were segmented by intensity thresholding. In order to limit the detection of the LAMP2 FITC signal inside lysosomes, as opposed to the cytoplasm, a 2D deconvolution procedure on the FITC channel was performed for reduction of the background noise. A sub-object was created to detect all the FITC signal in a modified mask starting 5 pixels from the edge of a nucleus inside the main mask with a width of 25 pixels (approximately 4 µm) resulting in a ring in the close vicinity of the nucleus (ring 1). A second sub-object was created starting 20 pixels away from the nucleus with a width of 75 pixels to detect the FITC signal in the rest of the cell (ring 2). The integrated FITC pixel intensities in ring 1 were divided by the integrated FITC pixel intensities in ring 1+ ring 2, resulting in a fraction of the FITC signal representing lysosomes in each cell that were present in the vicinity of the nucleus.

## Supplementary Figures

Supplementary Fig. 1, Bredahl Hansen et al. 2020

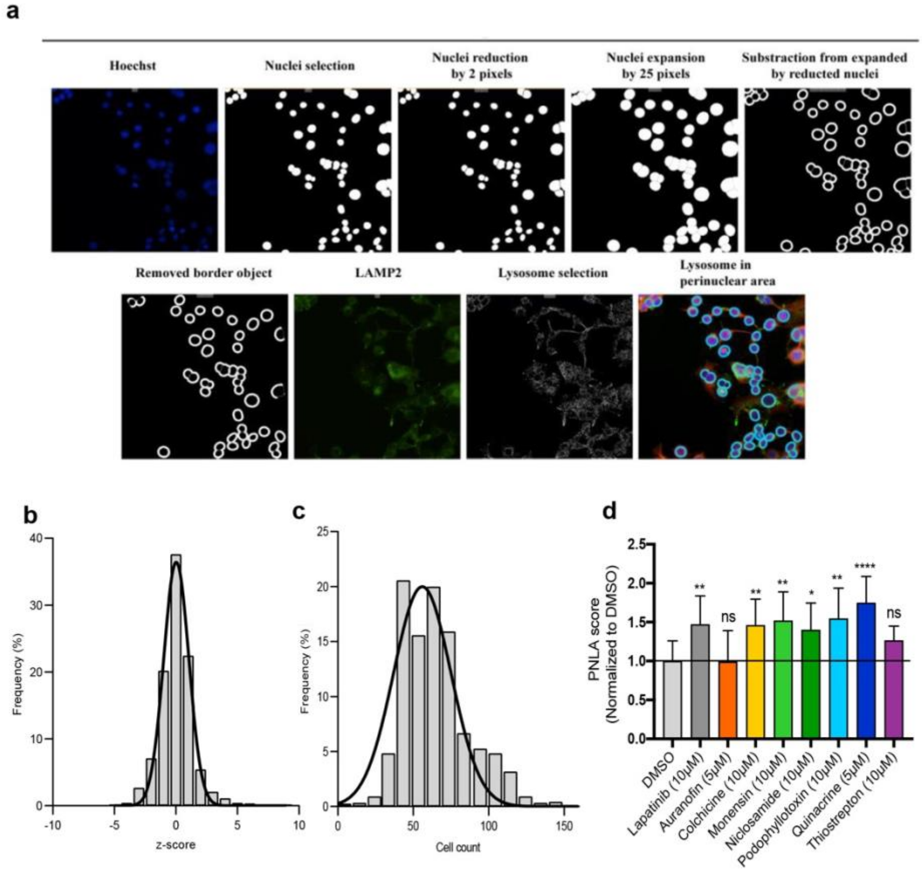


Sup.Fig.1: Demonstration of the Custom Module Operation Procedure set up with MetaXpress software to evaluate PNLA scores for the library screening and hit analysis and the histogram and Gaussian distributions for the z-scores and cell numbers from the screen. (**a**) MetaXpress Custom Module Operation Procedure. Top row: A schematic illustration of the algorithmic operations used to quantify the appearance of perinuclear lysosomes to define the PLNA score, and the bottom row: A visual presentation of the Custom Module setup from the MetaXpress. The illustrations and images show how the nucleus was detected (Hoechst stain) at DAPI channel, selected and reduced by 2 pixels (0.325 μm), after which it is expanded by 25 pixels (4,0625 μm). The reduced nuclei were subtracted from the expanded nucleus to create a “perinuclear ring”. The border objects were excluded and LAMP2 puncta were detected at the FITC channel. A logical operation was set to count the number of lysosomal puncta, average size of LAMP2 puncta per cell and average area covered by LAMP2 puncta inside the created perinuclear ring. (**b**) Histogram distribution of the z-score. Block interval is 1. (**c**) Histogram distribution of cell count per treatment. Block interval is 10. (**d**) Lysosome distribution in SKOV3ip1 cells upon 24-treatment with indicated drugs. Staining and detection was done as in 1**d**.

Supplementary Fig. 2,   Bredahl Hansen et al. 2020

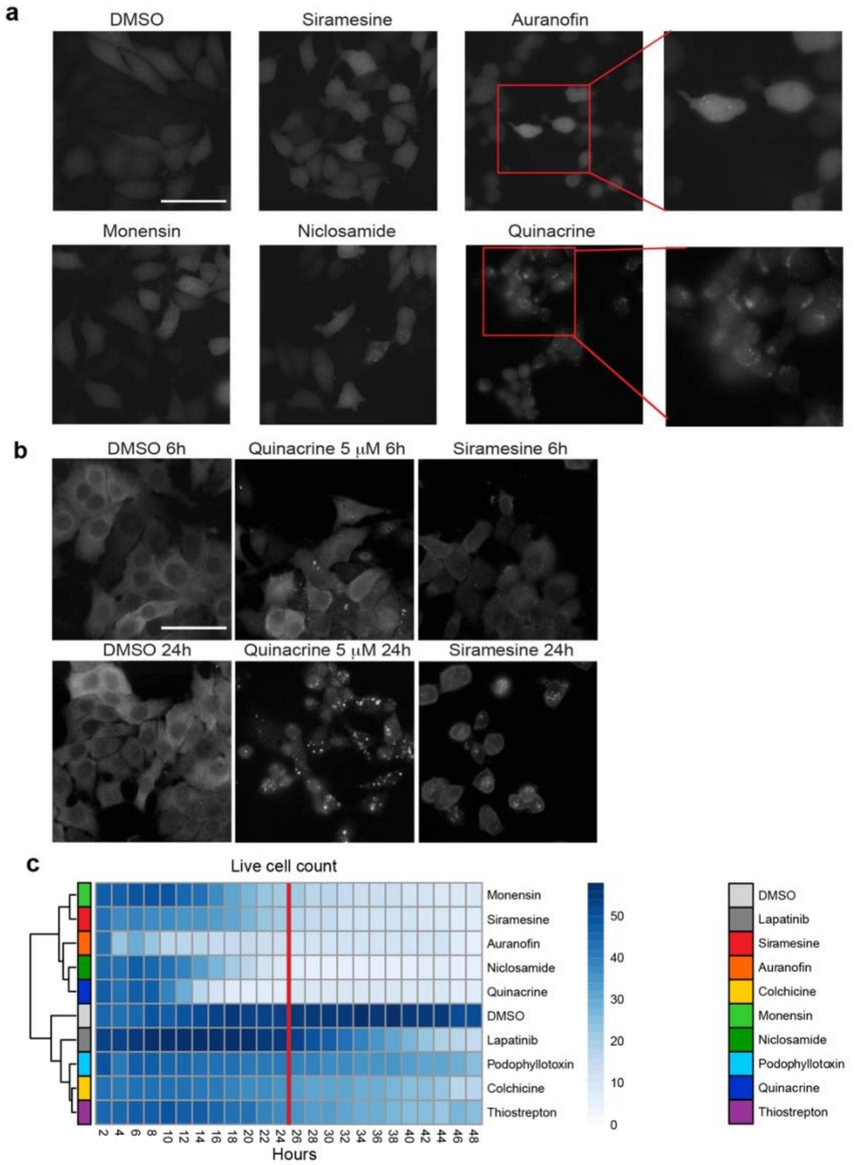


Sup.Fig. 2: Fluorescence images of indicated drug treatments (5 μM) of EGFP-Gal3 MCF7 and parental MCF7 cells for the detection of Gal3 puncta formation representing the quantifications for Fig 2**a** and 2b and presentation of the live cell counts corresponding the heatmap in 2**c**. (**a**) Representative images used for the detection of EGFP-Gal3 puncta in EGFP-Gal3 MCF7 cells (Fig 2**a**) for the assessment of LMP at the 12 h time point for the cells showing clear green puncta formation. White (light) dots represent EGFP-Gal3 puncta/dye visible in the green fluorescent (FITC) channel. 40x magnification. Scalebar is 55 μm. Red squares show the corresponding magnification (2x) areas. (**b**) Visualization of Gal3 puncta in MCF7 cells treated with indicated drugs, fixed and stained with Gal3 antibody at 6 h timepoint and at 24 h timepoint. 40x magnification. Scalebar is 55 μm. (**c**) The results corresponding live cell count are shown in Figure 2**c**. Heatmap presentation of the percentage of live cells was calculated from the total amount of cells detected in each site. The dead cell detection was done first by detecting high intensity nuclear stain signal (shrinking nucleus) and secondly by using transmitted light images. The cells not scoring as dead cells were calculated as live cells. The mean represents a mean of three biological repeats done in triplicate wells.

Supplementary Fig.3, Bredahl Hansen et al. 2020

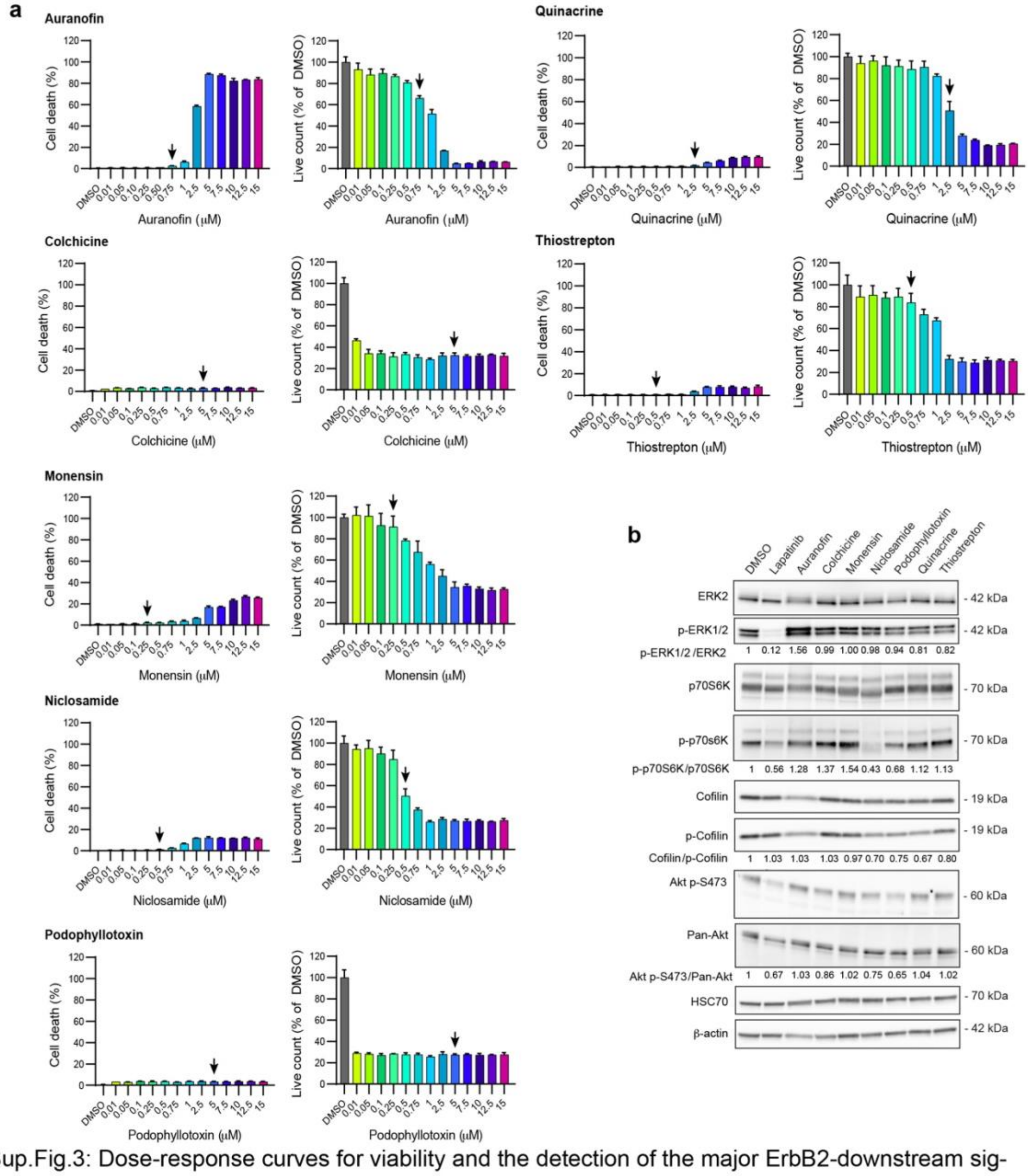


Sup.Fig.3: Dose-response curves for viability and the detection of the major ErbB2-downstream signalling pathway activation status for hit compounds. (**a**) Cells were treated with indicate drugs and concentrations and the viability (cell death and live cell count) was determined using Hoechst and Propidium Iodide staining 48 h after the indicated treatments. The dead (left side graphs) and live (right side graphs) cells were identified, measured and quantified using Celigo (n=1). (**b**) Immunoblots of p95-ErbB2-MCF7 cells treated with the lowest efficient drug concentrations (as in 3**a**) for 90 min for the detection of the activation status of the main ErbB2 downstream signaling pathways. ErbB2 downstream kinases (ERK2 and Akt) and their activation based on their phosphorylation status or (mTOR and PAK4) based on the phosphorylation status of their commonly used substrates p70S6K and cofilin, respectively as evaluated with immunoblotting. Each phosphor form was compared to the non-phosphorylated form of the protein. HSC70 and β-actin were used as loading controls. Quantifications of the blots are made with Image J and presented below the corresponding blot images.

Supplementary Fig. 4, Bredahl Hansen et al. 2020

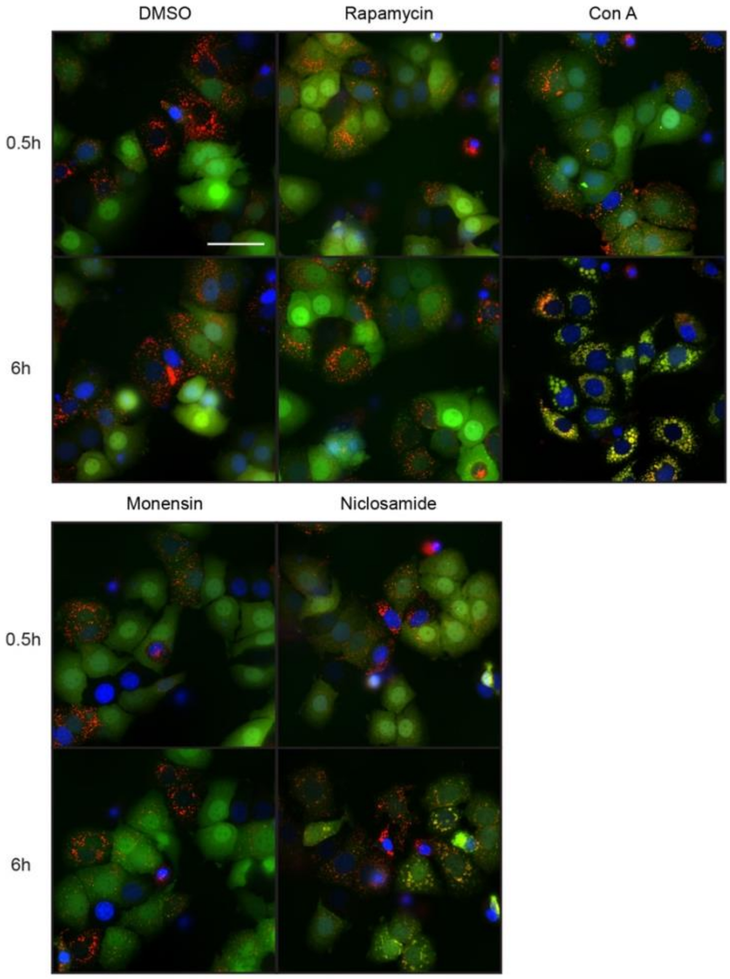


Sup.Fig.4: Representative images of autophagic flux measurements by tandem fluorescent live-cell assay for Fig 4b. Images of autophagic flux measurements of drug-treated tfLC3-MCF7 cells. Cells were treated with the lowest efficient concentrations of the indicated drugs for the quantification shown in Figure 4b. 2 nM ConA and 1μM Rapamycin were used as positive controls. The images were acquired at 0,5 h and 6 h after the treatment using the ImageXpress microconfocal system with 60x objective. Representative images of 0,5 h and 6 h timepoints are depicted for vehicle control (DMSO), increased autophagy control (Rapamycin), increased lysosomal pH control (Con A), lapatinib, and indicated compounds. Red color defines autophaglysosomes and yellow autophagosomes. Scalebar is 55 μm.

## References for supplementary information

1 Egeblad M, Mortensen OH, Jaattela M. Truncated ErbB2 receptor enhances ErbB1 signaling and induces reversible, ERK-independent loss of epithelial morphology. *International journal of cancer Journal international du cancer* (Research Support, Non-U.S. Gov't) 2001; 94: 185-191.

2 Brix DM, Rafn B, Bundgaard Clemmensen K, Andersen SH, Ambartsumian N, Jaattela M *et al*. Screening and identification of small molecule inhibitors of ErbB2-induced invasion. *Mol Oncol* 2014; 8: 1703-1718.

3 Strauss R, Li ZY, Liu Y, Beyer I, Persson J, Sova P *et al*. Analysis of epithelial and mesenchymal markers in ovarian cancer reveals phenotypic heterogeneity and plasticity. *PLoS One* 2011; 6: e16186.

4 Aits S, Kricker J, Liu B, Ellegaard AM, Hamalisto S, Tvingsholm S *et al*. Sensitive detection of lysosomal membrane permeabilization by lysosomal galectin puncta assay. *Autophagy* 2015; 11: 1408-1424.

5 Kimura S, Noda T, Yoshimori T. Dissection of the autophagosome maturation process by a novel reporter protein, tandem fluorescent-tagged LC3. *Autophagy* 2007; 3: 452-460.

6 Szyniarowski P, Corcelle-Termeau E, Farkas T, Hoyer-Hansen M, Nylandsted J, Kallunki T *et al*. A comprehensive siRNA screen for kinases that suppress macroautophagy in optimal growth conditions. *Autophagy* 2011; 7: 892-903.

7 Tvingsholm SA, Hansen MB, Clemmensen KKB, Brix DM, Rafn B, Frankel LB *et al*. Let-7 microRNA controls invasion-promoting lysosomal changes via the oncogenic transcription factor myeloid zinc finger-1. *Oncogenesis* 2018; 7: 14.
