# Supplementary material for: Identification of lysosome‐targeting drugs with anti‐inflammatory activity as potential invasion inhibitors of treatment resistant HER2 positive cancers
Source: Cell Oncol (Dordr). 2021 May 3;44(4):805–20. doi: 10.1007/s13402-021-00603-2 (PMC8090911; doi:10.1007/s13402-021-00603-2)
Supplement: Supplementary file 3 — (PDF 5.06 MB) [file 13402_2021_603_MOESM3_ESM.pdf]

Bredahl Hansen et al. 2020 LC3 blots and membranes

LC3B and Hsp90

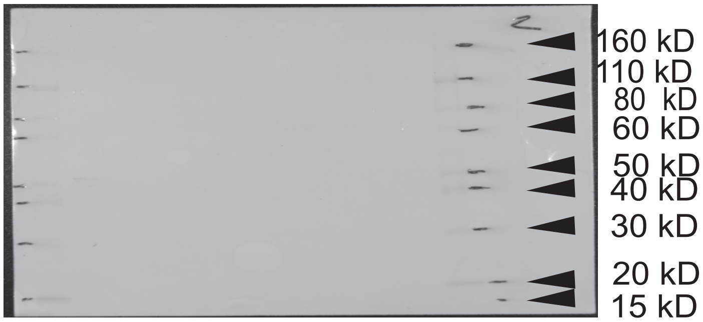

membrane

LC3B

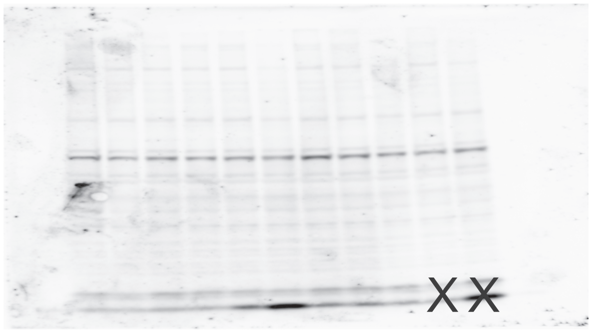

exposure

Hsp90

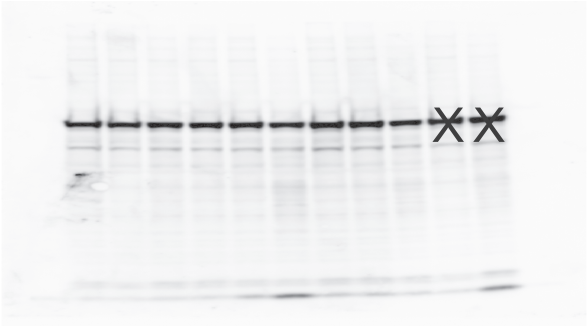

exposure

Additional blots used for quantification of LC3B:

LC3B

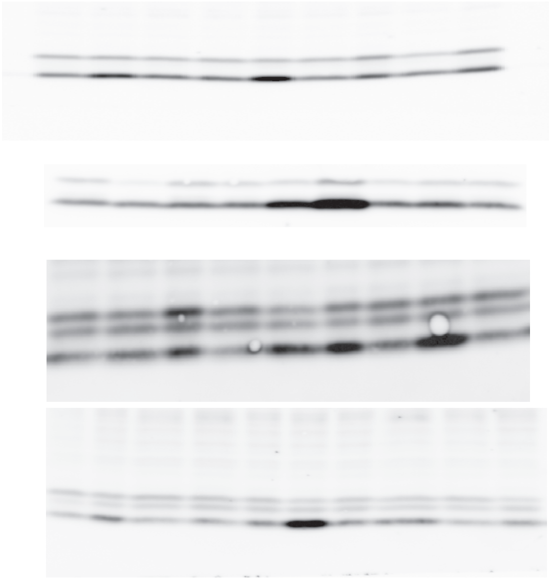

HSP90

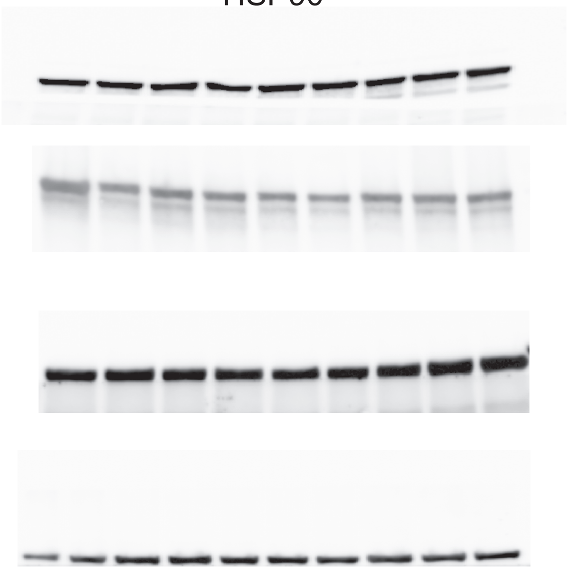

Immunoblots for LC3-II and its loading control HSP90. Corresponding membranes and un-cut blots for the images shown in Figure 4a. Additional blots used in quantification are shown in the bottom of the figure. Samples are loaded in the same order as in the Figure 4a. X covers additional samples not use din this study.

Bredahl Hansen et al. 2020 SQSTM blots and membranes

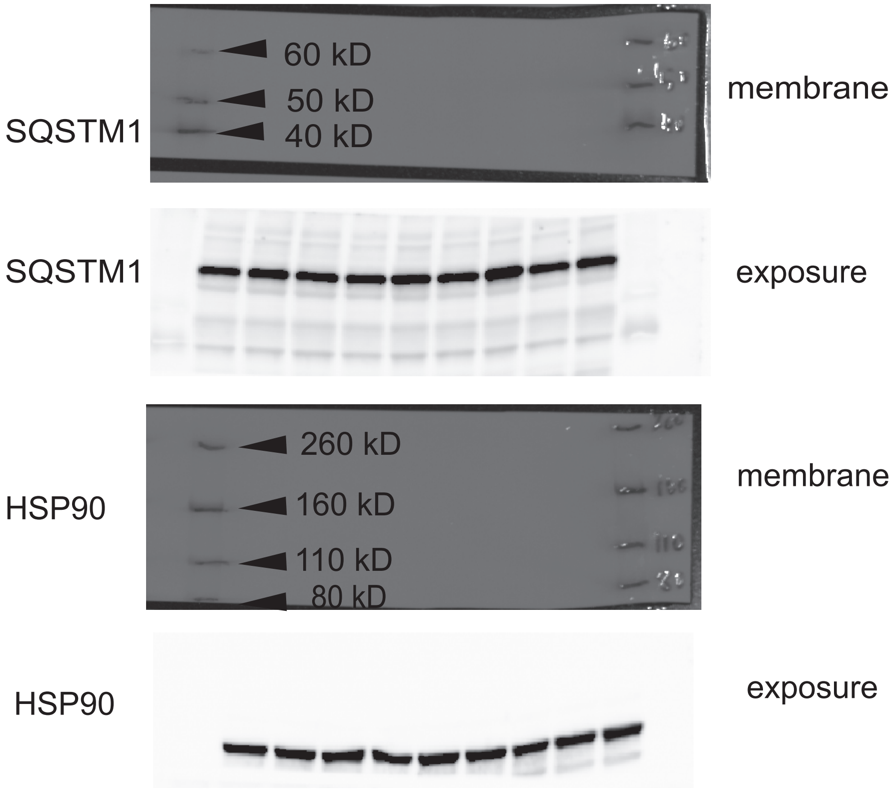

Additional blots used for quantification of SQSTM1:

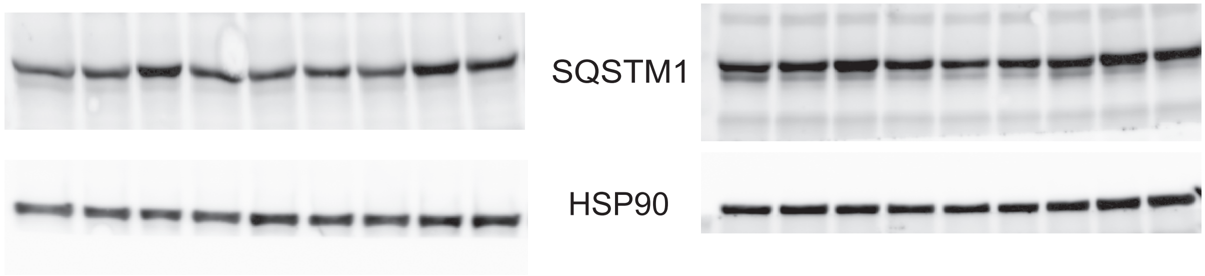

Immunoblots for SQSTM and its loading control HSP90. Images of corresponding membranes and un-cut blots shown in Figure 4a. Additional blots used in quantification are shown in the bottom of the figure. Samples are loaded in the same order as in the Figure 4a.

Bredahl Hansen et al. 2020 LAMP2 blots and membranes

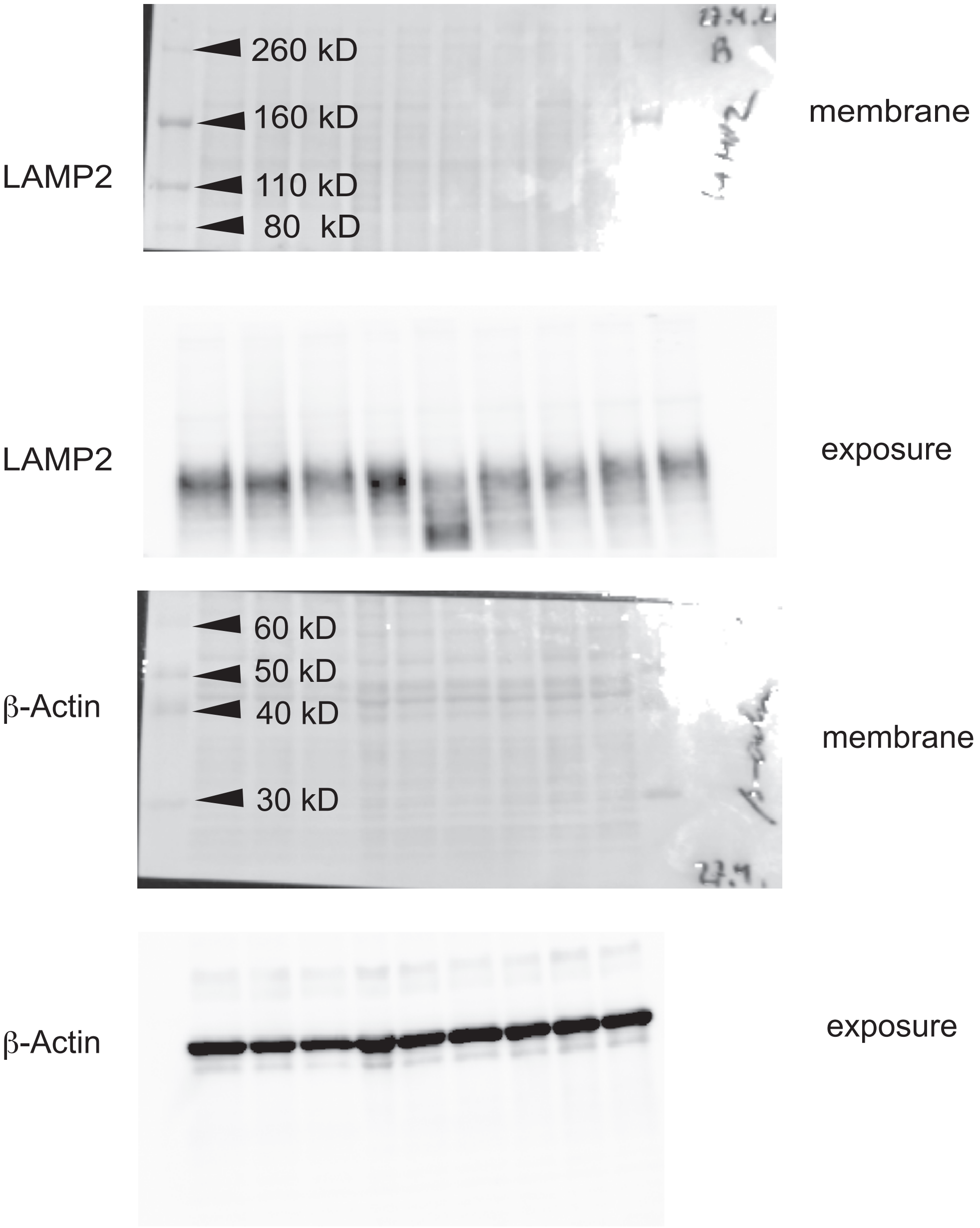

Additional blots used for quantification of LAMP2:

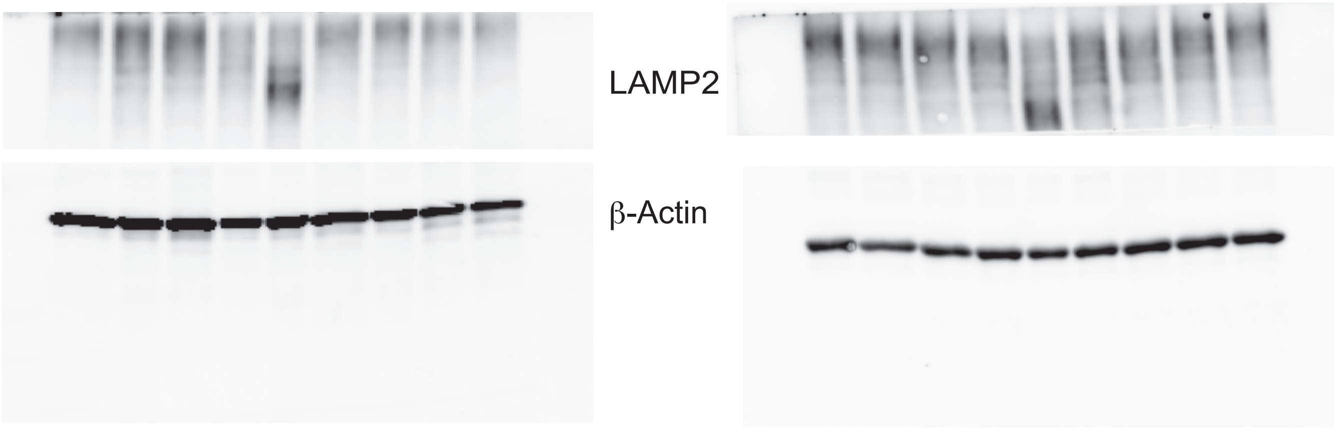

Immunoblots for LAMP2 and β-actin. Corresponding membranes and un-cut blots for the images shown in Figure 3c. Additional blots used in the quantification are shown in the bottom of the figure. Samples are loaded in the same order as in the Figure 3c.

Bredahl Hansen et al. 2020 Cathepsin B blots and membranes

Cathepsin B

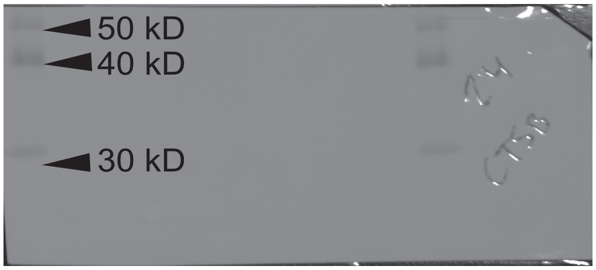

membrane

Cathepsin B

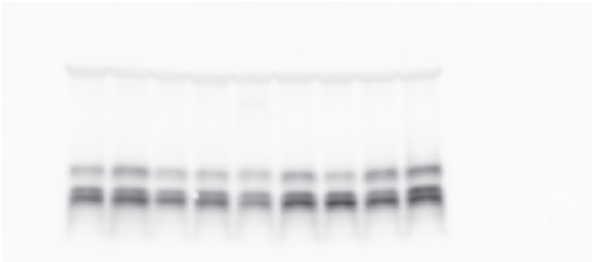

exposure

β-Actin

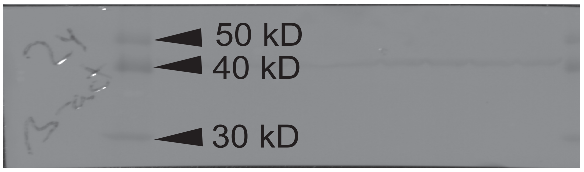

membrane

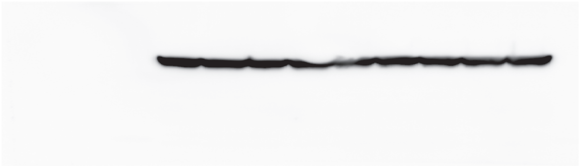

exposure

β-Actin

Additional blots used for quantification of Cathepsin B:

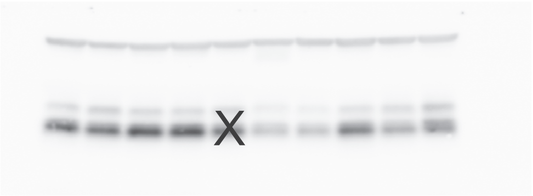

Cathepsin B

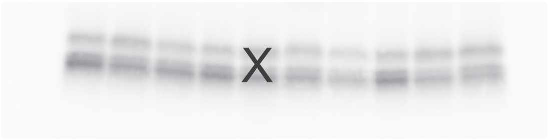

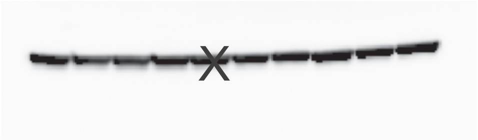

β-Actin

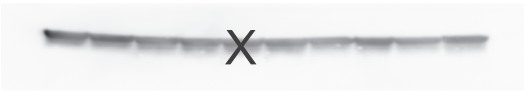

Immunoblots for cathepsin B and β-actin. Corresponding membranes and un-cut blots for the images shown in Figure 4c. Additional blots used in the quantification are shown in the bottom of the figure. X covers an additonal sample not used in this study.
