# Supplementary material for: Identification of lysosome‐targeting drugs with anti‐inflammatory activity as potential invasion inhibitors of treatment resistant HER2 positive cancers
Source: Cell Oncol (Dordr). 2021 May 3;44(4):805–20. doi: 10.1007/s13402-021-00603-2 (PMC8090911; doi:10.1007/s13402-021-00603-2)
Supplement: Supplementary file 4 — (PDF 4.07 MB) [file 13402_2021_603_MOESM4_ESM.pdf]

Bredahl Hansen et al. 2020 Akt and p-Akt blots and membranes

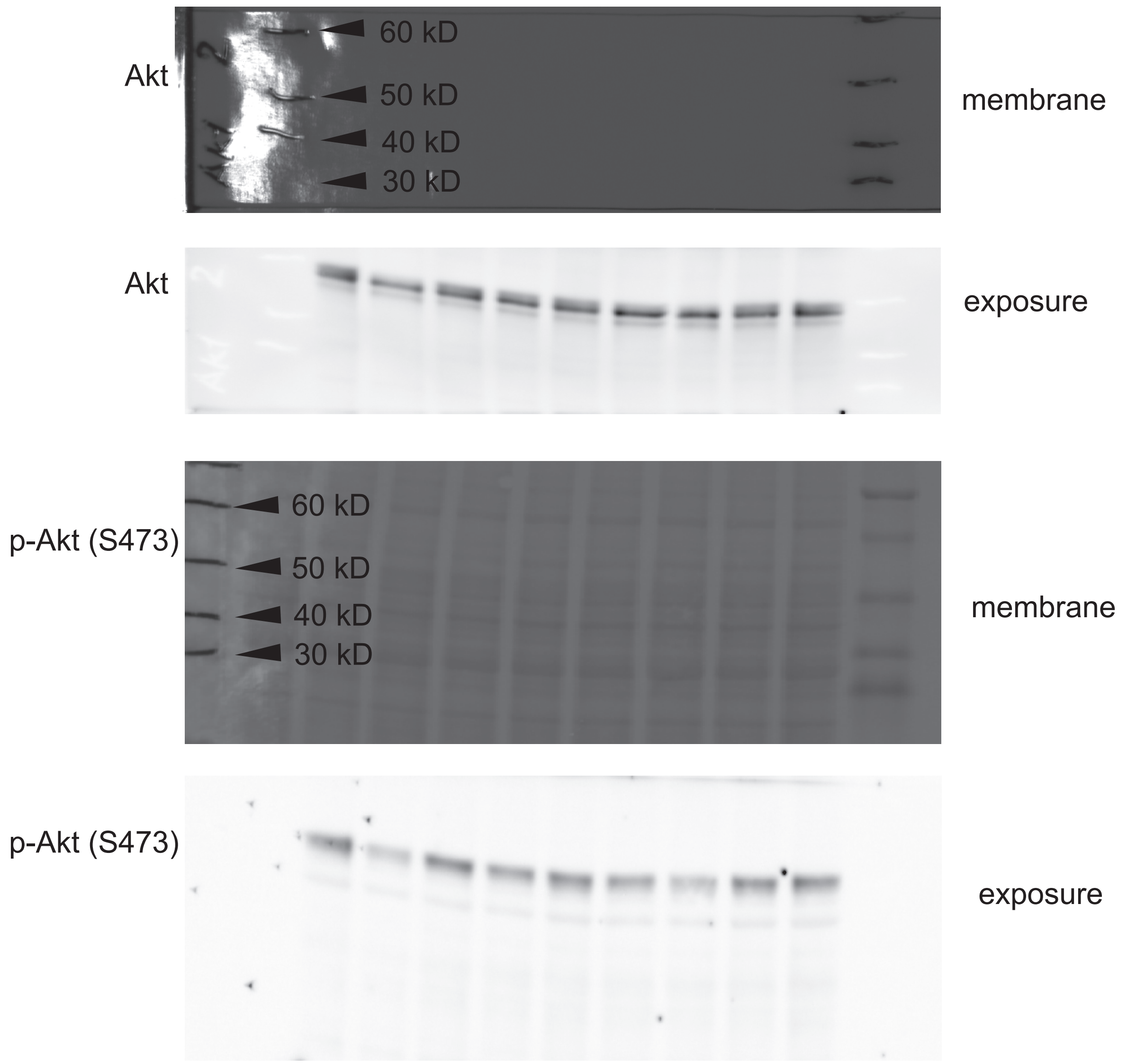


Immunoblots for phosphor phosphor AKT and AKT. Corresponding membranes and un-cut blots for the images shown in Figure S3b.

Bredahl Hansen et al. 2020 Cofilin and p-Cofilin blots and membranes

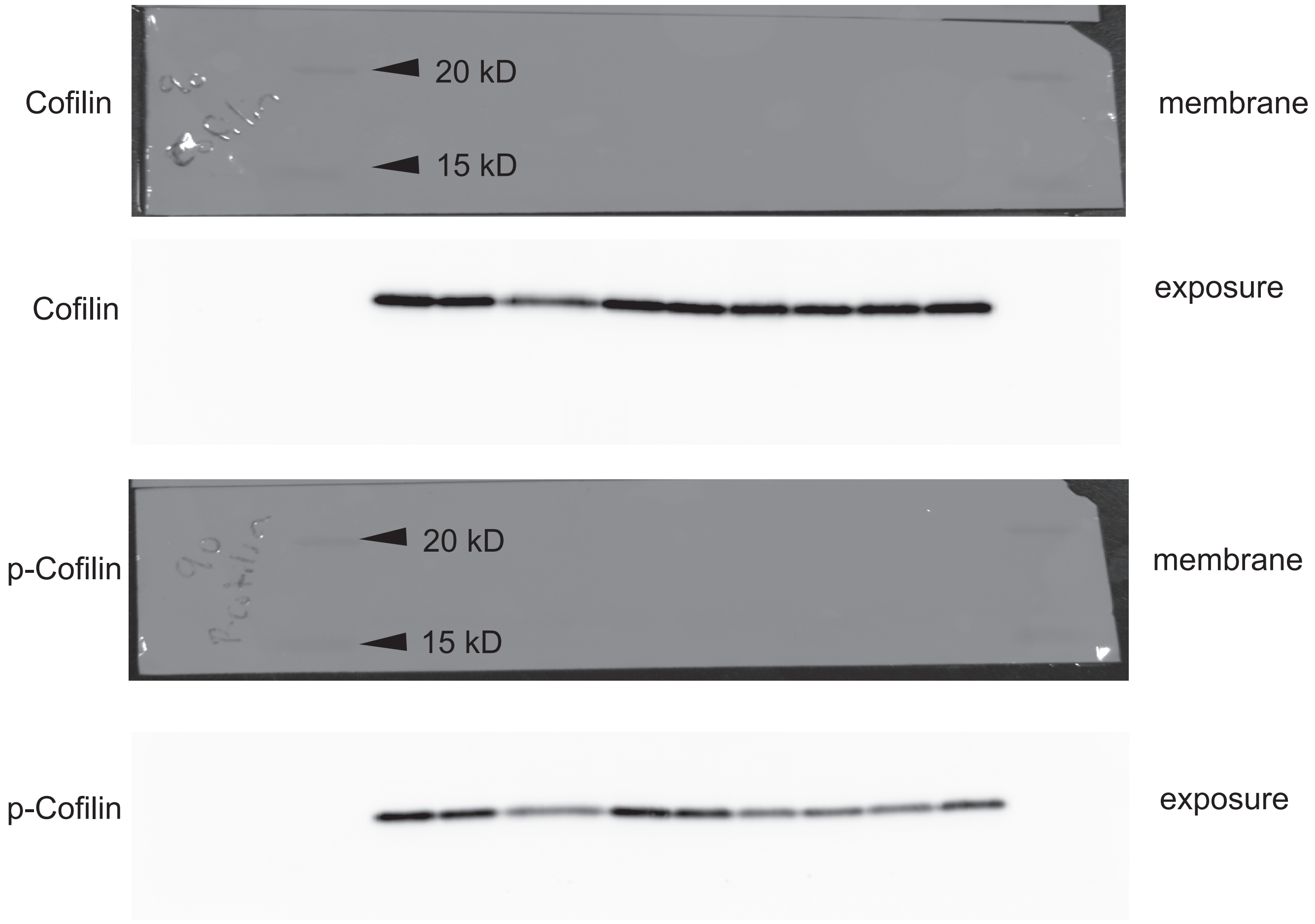


Immunoblots for phosphor Cofilin and Cofilin. Corresponding membranes and un-cut blots for the images shown in Figure S3b.

Bredahl Hansen et al. 2020 ERK2 and pERK blots and membranes

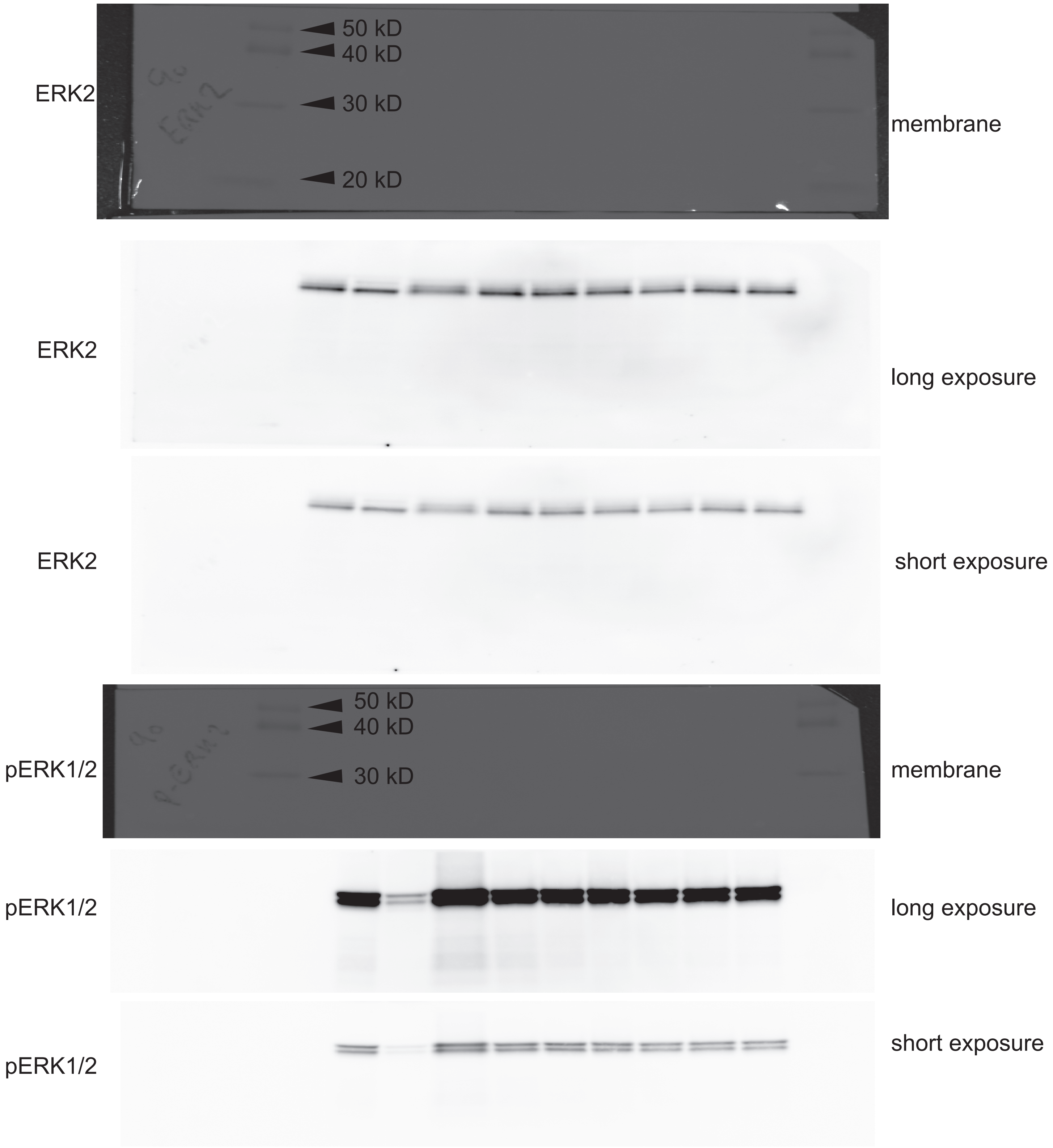


Immunoblots for phorphor ERK and ERK2. Corresponding membranes and un-cut blots for the images shown in Figure S3b.

Bredahl Hansen et al. 2020 p70S6K and p-p70S6K blots and membranes

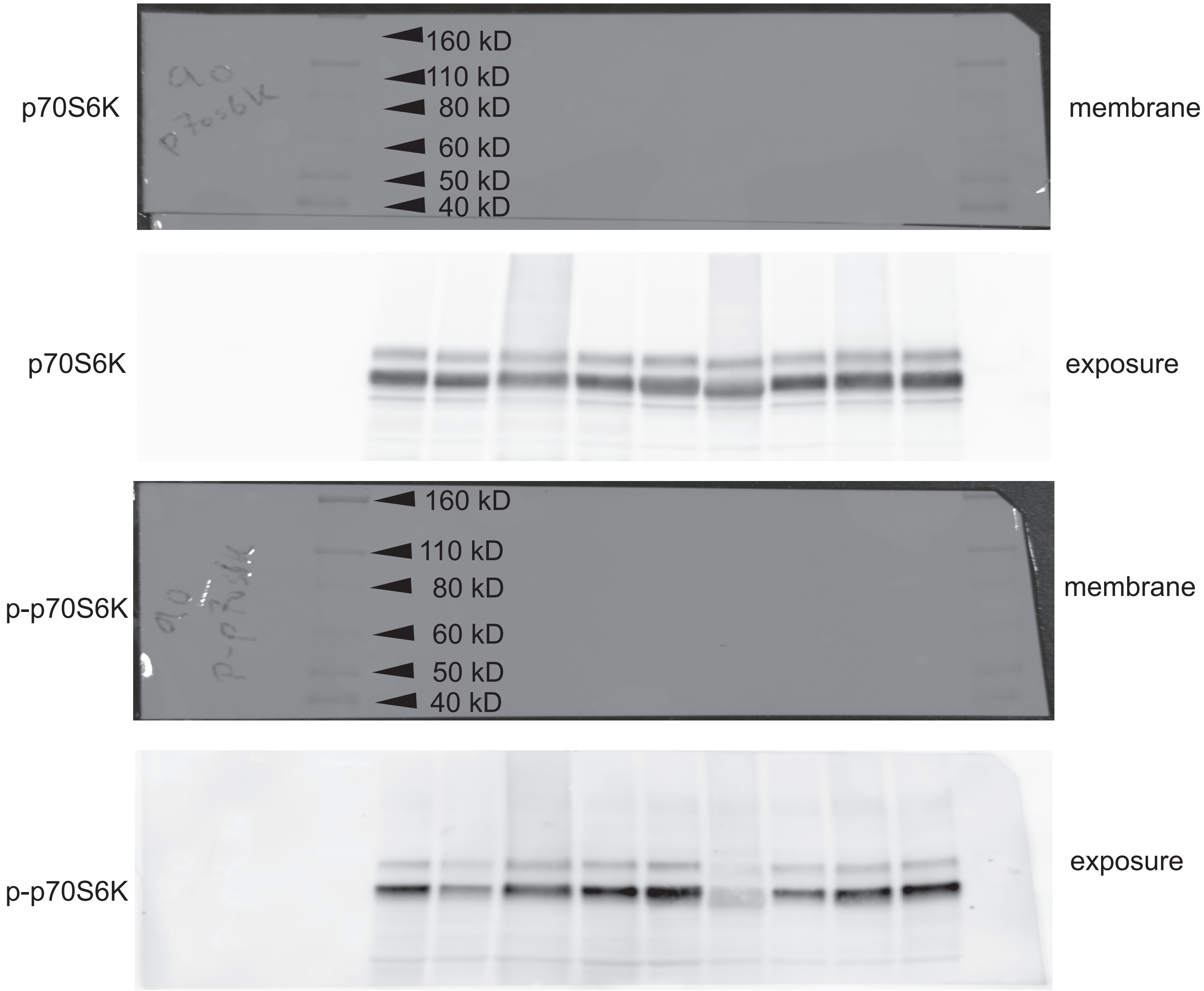


Immunoblots for phosphor p70S6K and p70S6K. Corresponding membranes and un-cut blots for the images shown in Figure S3b.
